# Rapid Divergence Followed by Adaptation to Contrasting Ecological Niches of Two Closely Related Columbine Species *Aquilegia japonica* and *A. oxysepala*

**DOI:** 10.1093/gbe/evz038

**Published:** 2019-02-21

**Authors:** Ming-Rui Li, Hua-Ying Wang, Ning Ding, Tianyuan Lu, Ye-Chao Huang, Hong-Xing Xiao, Bao Liu, Lin-Feng Li

**Affiliations:** 1 Ministry of Education Key Laboratory for Biodiversity Science and Ecological Engineering, School of Life Sciences, Fudan University, Shanghai, China; 2 Key Laboratory of Molecular Epigenetics of the Ministry of Education (MOE), Northeast Normal University, Changchun, China; 3 McGill University and Genome Quebec Innovation Center, Montreal, Quebec, Canada

**Keywords:** *Aquilegia*, adaptation, ecological specialization, selection, speciation

## Abstract

Elucidating the mechanisms underlying the genetic divergence between closely related species is crucial to understanding the origin and evolution of biodiversity. The genus *Aquilegia* L. has undergone rapid adaptive radiation, generating about 70 well-recognized species that are specialized to distinct habitats and pollinators. In this study, to address the underlying evolutionary mechanisms that drive the genetic divergence, we analyzed the whole genomes of two ecologically isolated *Aquilegia* species, *A. oxysepala* and *A. japonica* as well as their putative hybrid. Our comparative genomic analyses reveal that while the two species diverged only recently and experienced recurrent gene flow, a high level of genetic divergence is observed in their nuclear genomes. In particular, candidate genomic regions that show signature of selection differ dramatically between the two species. Given that the splitting time of the two species is broadly matched with the decrease in effective population sizes, we propose that allopatric isolation together with natural selection have preceded the interspecific gene flow in the process of speciation. The observed high genetic divergence is likely an outcome of combined effects of natural selection, genetic drift and divergent sorting of ancestral polymorphisms. Our study provides a genome-wide view of how genetic divergence has evolved between closely related species.

## Introduction

Unravelling the evolutionary mechanisms whereby the species arise and adapt to diverse environments is fundamental to understanding the origin and persistence of biodiversity (Schluter 2000; Coyne and Orr 2004; Nosil 2012). A critical step in elucidating this issue is to determine how the genetic divergence evolved between incipient species and to what extent it caused the occurrence of reproductive isolation (RI) (Coyne and Orr 2004; Gavrilets 2004; Nosil and Feder 2012). In general, the establishment of RI can be driven by either natural selection acting on locally adaptive alleles (extrinsic reproductive isolation) or by the evolution of genetic incompatibilities through genetic mutation, random drift, and genomic conflict (intrinsic reproductive isolation) (Crespi and Nosil 2013; Seehausen et al. 2014; Trucchi et al. 2017). In the model of speciation with gene flow, three fundamental mechanisms can promote the evolution of RI (Feder et al. 2012, 2013). The initial stage of genetic divergence is initiated by natural selection directly acting on the loci correlated with environmental differences or mate choice (Feder et al. 2013; Riesch et al. 2017). Then, the combination of divergence hitchhiking and genome hitchhiking may further promote the magnitude and spread of “genomic islands of divergence” across the genome (Feder et al. 2012, 2013). Genetic evidence supporting this “genic model of speciation” (Wu 2001) was reported in diverse animals (e.g., stickleback, Darwin’s finch, and cichlid) and plants (e.g., monkey flower and Arabidopsis) (Hancock et al. 2011; Arnegard et al. 2014; Lamichhaney et al. 2015; Malinsky et al. 2015; Ferris 2016). On the other hand, reproductive barriers can also evolve in the absence of gene flow through rare founder effects or rapid evolution of RI associated with standing and/or novel variations (Mayr 1963; Barton and Charlesworth 1984; Gavrilets 2004; Kirkpatrick and Barton 2006; Nosil et al. 2017). In the geographic speciation model, for example, genetic divergence can arise rapidly as a result of geographic isolation and random genetic drift (Wright 1943; Slatkin 1993). These empirical studies suggest that RI can evolve in the presence or absence of gene flow due to the combination of different evolutionary mechanisms.

The genus *Aquilegia* L. is a model system to study the genetic basis underpinning adaptive radiation and floral development (Hodges and Derieg 2009; Kramer 2009; Kramer and Hodges 2010; Sharma et al. 2014). This genus encompasses ∼70 recently diversified species that are distributed in the temperate zones of northern hemisphere and specialized to distinct ecological habitats and pollinators (Munz 1946; Hodges and Arnold 1994; Nold 2003; Kramer and Hodges 2010). Phylogenetic and biogeographic analyses revealed Eastern Asia origin of the *Aquilegia* species, followed by two independent adaptive radiations in North America and Western Eurasia, respectively (Bastida et al. 2010; Fior et al. 2013). In North American columbines, floral or flower morphologies and flower colors are specialized to different pollinators (Hodges and Derieg 2009). For example, the changes in spur length and flower orientation are highly correlated with the shifts of pollinators from bee to hummingbird and hummingbird to hawkmonth (Fulton and Hodges 1999; Hodges et al. 2002; Whittall and Hodges 2007). Genetic dissection of these floral traits revealed that nucleotide substitutions and expression changes of genes involved in the flavonoid pathway (e.g., *CHS* and *ANS*) and cell division (e.g., *KNOX* and *APETALA3*) are tightly associated with the floral morphological differences (Hodges and Derieg 2009; Sharma and Kramer 2013; Yant et al. 2015). Among the European columbines, climatic oscillations during the Quaternary period resulted in the formation of multiple geographically and ecologically isolated species (Fior et al. 2013). In contrast, while >20 morphologically distinct species are identified in Asia, evolutionary mechanisms underlying the diversification of these species remain largely unclear. Fior *et al.* (2013) proposed that the central Asian species share the most recent common ancestor (MRCA) with the European columbines, and the North Asian species are established through a back-to-Asia recolonization from the European stock. In northeastern Asia, five well-recognized species are found, of which the species *A. amurensis* and *A. parviflora* are restricted to the northern Greater Khingan Mountains and phylogenetically close to the North American columbine species; the remaining three species, *A. viridiflora*, *A. oxysepala*, and *A. japonica*, share the MRCA with North Asian and European columbine species (Fior et al. 2013). Of significance, the species pair, *A. japonica* and *A. oxysepala*, are closely related species that inhabit the alpine tundra and low altitude forest niches, respectively (Li et al. 2011, 2014). Our previous study demonstrated that although the two species diverged from each other recently and experienced gene flow during the adaptation process, high genetic divergence was observed at the analyzed loci in the nuclear genome (Li et al. 2014). However, these observations mainly relied on a limited number of chloroplast and nuclear loci, while the genome-wide patterns of genetic divergence and natural selection remained uninvestigated.

In this study, a total of 32 accessions of the species *A. oxysepala* and *A. japonica* were selected according to their geographical distribution and genetic background. Our previous study identified putative hybrids in the contact zone of the two species (Li et al. 2014). Here, we sequenced the genomes of two accessions to assess genetic dynamics of the parental genomes in the hybrid. In addition, Filiault *et al.* (2018) proposed that the two allopatric species, *A. japonica* and *A. sibirica*, are derived from the MRCA but the former species recently hybridized with a phylogenetically distinct but sympatrically distributed species, *A. oxysepala*. To further test this speciation by hybridization model at the population level, we combined our genomic data with those from five additional *Aquilegia* accessions (*A. oxysepala*, *A. japonica*, *A. sibirica*, *A. vulgaris*, and *A. coerulea*) generated from Filiault *et al.* (2018). The availability of these genomic data provides an opportunity to address the demographic histories of these *Aquilegia* species at the genome-wide scale. Specifically, comparative genomic analyses of the *A. oxysepala* and *A. japonica* can shed light on the evolutionary mechanisms that have promoted the genetic divergence.

## Materials and Methods

### Plant Materials and DNA Extraction

In our previous study, a total of 287 samples were collected to represent the current geographic distributions of *A. japonica*, *A. oxysepala* and their hybrids in northeastern China (Li et al. 2014). Here, 32 representative accessions of the two species (16 *A. oxysepala* and 16 *A. japonica*, respectively) and two hybrids were selected (supplementary table S1, Supplementary Material online). Genomic DNAs of the 34 samples were extracted from silica gel-dried mature leaves using the TianGen plant genomic DNA Kit (TianGen, Tianjin, China). Whole genome data of the four *Aquilegia* species, *A. vulgaris* (SRR414349), *A. sibirica* (SRR415090), *A. oxysepala* (SRR414321), and *A. japonica* (SRR414399), were downloaded from the documented study of Filiault *et al.* (2018) at GenBank. Genome assembly of the reference species *A. coerulea* was obtained from Phytozome (https://phytozome.jgi.doe.gov). Of the five downloaded *Aquilegia* species, *A. vulgaris*, and *A. coerulea* were used to represent the European and North American columbine species, respectively. The remaining three Asian columbine species (*A. sibirica*, *A. oxysepala*, and *A. japonica*) were employed to confirm their phylogenetic positions in the genus *Aquilegia*.

### Sequencing, Mapping, and Genotyping

Short-insert (350 bp) DNA libraries of the 34 *Aquilegia* samples collected in this study were constructed by Beijing Genomics Institute (BGI) in Shenzhen. Whole genome resequencing was performed using the Illumina Hiseq 2000 platform (Illumina, CA). All data generated in this study were submitted to GenBank under the Bioproject number PRJNA433751. Raw reads of all *Aquilegia* accessions were assessed by the program FastQC (Andrews 2010). Low quality reads (>20% positions of the read with base quality <30) were removed using NGStoolkit (Trivedi et al. 2014). Available chloroplast genome sequence (AC253869) of the species *A. coerulea* was downloaded from GenBank. The nuclear reference genome was obtained from Phytozome with the permission from Elena Kramer and Scott Hodges. All filtered reads were mapped against the chloroplast and nuclear reference genomes using BWA (Li and Durbin 2010) with the default parameters. Single nucleotide polymorphisms (SNPs) and insertions/deletions (INDELs) were reported using the program SAMtools (Li et al. 2009) with our previously used parameters (Li LF et al. 2017). Raw SNPs and INDELs were filtered using our custom Perl scripts with the cutoff “mapping quality (MQ) >20, read depth (RD) >3.” Missing data in the data matrix was determined according to our previous strategies (Jiang et al. 2018). All filtered variants were subjected to the subsequent data analyses.

### Phylogenetic and Population Structure Analyses

Incongruences between the nuclear and chloroplast phylogenies were reported in the genus *Aquilegia*, with the *A. oxysepala* and *A. japonica* sharing most of their chloroplast haplotypes (Li et al. 2014) but the former species possessing a nuclear genome distinct to all other congeneric species (Filiault et al. 2018). In this study, we employed the maximum likelihood (ML), neighbor-joining (NJ), and unweighted pair group method with arithmetic mean (UPGMA) methods to infer the phylogenetic relationships of the 39 *Aquilegia* accessions. Chloroplast and nuclear SNP matrices were generated according to our previous strategies (Li MR et al. 2017): *i*) only the SNPs that showed no missing data across all accessions were retained; *ii*) the SNP matrix was then converted into homozygous and heterozygous matrices using custom Perl scripts. The homozygous matrix only includes the SNPs that are homozygous across all accessions. In contrast, the heterozygous matrix contains both homozygous and heterozygous SNPs. NJ and UPGMA trees were constructed using MEGA 7.0 (Kumar et al. 2016) based on the homozygous and heterozygous matrices, respectively. The same SNP matrices were also employed to construct ML trees using RAxML (Stamatakis 2014) and to calculate pair-wise genetic distances using MEGA 7.0 (Kumar et al. 2016). Genetic assignments for each of these *Aquilegia* accessions were performed based on the nuclear genome data set using ADMIXTURE (Alexander et al. 2009). Putative genetic clusters (*K* values) were inferred from 1 to 10 with three independent iterations. The best genetic assignment was estimated by comparing the cross-validation error of each *K* value. Barplots of the resulting genetic assignments were illustrated using our custom R scripts.

### Nucleotide Variation Pattern and Genome-Wide Scanning of Selection

Nucleotide diversity (π), segregating site (*S*), and Tajima’s *D* were calculated for the *A. oxysepala* and *A. japonica* and their hybrid using VCFtools (Danecek et al. 2011), respectively. For the nuclear genome, we performed a genome-wide assessment with 100-kb nonoverlapping sliding window. In contrast, the chloroplast genome was treated as a single matrix due to its short length (∼129 kb). The phylogenetic and population structure analyses detailed above revealed spatial genetic structure of the two species, we therefore divided the 16 *A. oxysepala* accessions into southern and northern groups according to their geographic locations and genetic background. Similarly, the 16 *A. japonica* accessions were also defined as southern, middle and northern groups. Population parameters (π, *S*, and Tajima’s *D*) of the chloroplast and nuclear genomes were also calculated for the five groups separately. To evaluate if *A. oxysepala* and *A. japonica* share similar nucleotide variation patterns, we compared the nucleotide diversity (π) and Tajima’s *D* for each of the 100-kb sliding windows between the two species. Intra- and interspecific genetic differentiation (*F*_ST_) was also calculated for the two species using VCFtools (Danecek et al. 2011). The parameters fixation site (Fs) and absolute differentiation (Dxy) were estimated using Perl scripts. Distribution patterns of the nucleotide diversity (π) and genetic divergence (*F*_ST_ and Dxy) were visualized using the R package Circos (Gu et al. 2014). Significance of the overall nucleotide variation pattern (π and *F*_ST_) within and between the two species was assessed using *t*-test. Correlations of the nucleotide diversity and genetic differentiation for each 100-kb sliding window were evaluated at the group and species levels using the Spearman correlation coefficient (ρ). To identify the genomic regions under natural selection, we defined the top 5% lowest nucleotide diversity (LND) windows as the candidates and compared them with high interspecific genetic differentiation (HIGD) regions (top 5% windows). The candidate genomic regions that showed HIGD with LND in either of the two species were regarded as the targets of adaptive selection during the ecological specialization. Likewise, the genomic regions that showed HIGD with LND in both species are defined as the targets of divergent selection in the process of speciation. To further evaluate if selective sweep acts on the two species, we performed genome-wide scanning across the nuclear genome using SweeD (Pavlidis et al. 2013). Each chromosome was divided into 2,000 bins and presence or absence of a signature of selective sweep was determined by the value of composite likelihood ration (CLR) statistic (Nielsen et al. 2005). Manhattan plot of selective sweep was visualized for each species using the R package qqman (Turner 2014).

### Demographic History and Gene Flow

To infer the demographic histories of *A. oxysepala* and *A. japonica*, fluctuations in effective population size of the two species were simulated using SMC++ (Terhorst et al. 2017). Demographic histories of the three species (*A. sibirica*, *A. vulgaris*, and *A. coerulea*) were not estimated due to the limitation of available accessions used in this study. Input data were converted from the VCF into SMC++ input format using the command “smc++ vcf2smc.” Historical effective population sizes of the two species were estimated with the command “smc++ estimate.” The average mutation rate of the nuclear genome was set to 10^−8^ per bp per generation. To estimate the splitting time between the *A. oxysepala* and *A. japonica*, we simulated the joint frequency spectrums for both species using one year as generation time. Then, we refined the marginal distribution into the joint demography using the command “smc++ split.” All results were visualized using the command “smc++ plot.” As a complementary, we also estimated gene flow among these *Aquilegia* species using Treemix (Pickrell and Pritchard 2012). Population ancestries of these species were presented using a ML tree and only the top five migration events were visualized. To assess the degrees of genetic similarity among *A. sibirica*, *A. oxysepala*, and *A. japonica*, we identified the shared and species-specific variants (SNPs and INDELs) at both the chromosome and whole-genome levels, respectively. Information of the SNPs and INDELs was extracted from the VCF data set using Perl scripts and visualized using the R package VennDiagram (Chen and Boutros 2011).

## Results

### Sequence Polymorphism and Allele Frequency

**Fig. 1.**
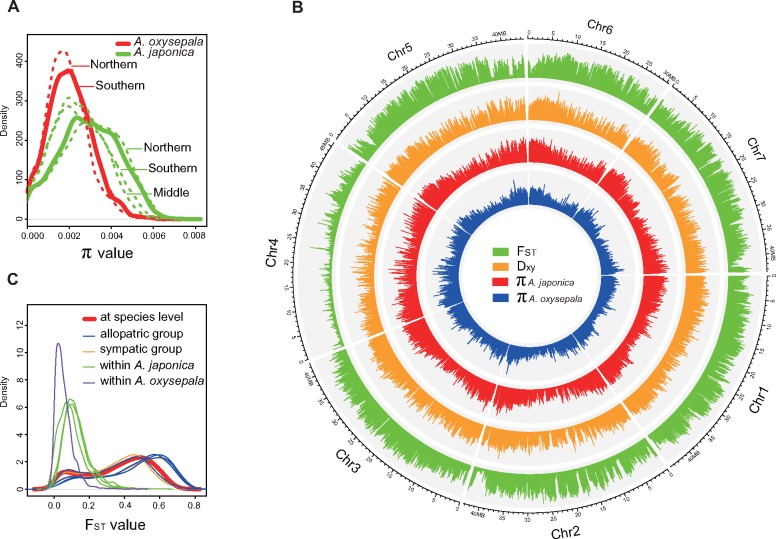
—Nucleotide diversity (π) and genetic divergence (*F*_ST_ and Dxy) of the *Aquilegia japonica* and *A. oxysepala* at the group and species levels. (*A*) The dashed and solid lines represent the nucleotide diversity (π) for each group and species of *A. japonica* (green) and *A. oxysepala* (red), respectively. (*B*) The four layers from outside to inner are the genetic differentiation (*F*_ST_), absolute divergence (Dxy), and nucleotide diversity (π) for the *A. oxysepala* and *A. japonica*, respectively. (*C*) The bolded red line represents the genetic divergence (*F*_ST_) between the *A. oxysepala* and *A. japonica* at the species level. Blue and orange colors are the genetic divergence between the allopatric and sympatric groups of the two species. Green and purple colors represent the intraspecific genetic differentiation within the *A. oxysepala* and *A. japonica*, respectively.

Nucleotide diversity (π and *S*) of the chloroplast and nuclear genomes was calculated for the two species and their hybrid separately. At the species level, *A. japonica* (π = 0.002968 and *S* = 3,647,897) possesses obviously higher nucleotide diversity relative to the *A. oxysepala* (π = 0.002074 and *S* = 2,370,500) at the nuclear genome (table 1). It is surprising that although only two accessions were included, similar level of nucleotide diversity (π = 0.003626 and *S* = 1,927,265) is found in the hybrid. At the group level, the southern group of *A. oxysepala* exhibits relatively higher nucleotide diversity (π = 0.002068 and *S* = 2,045,488) compared to the northern group (π = 0.001907 and *S* = 1,582,564) (table 1). In contrast, the northern group of *A. japonica* (π = 0.003094 and *S* = 2,700,605) harbors the highest level of nucleotide diversity than those of the southern (π = 0.002535 and *S* = 2,151,279) and middle (π = 0.002434 and *S* = 1,725,215) groups (table 1). A similar phenomenon is also observed at the chloroplast genome where *A. japonica* (π = 0.000311 and *S* = 174) preserves higher nucleotide diversity compared to *A. oxysepala* (π = 0.00255 and *S* = 144) and hybrid (π = 0.000261 and *S* = 65) (table 1). We also calculated the nucleotide diversity for each 100-kb sliding window for the two species. While *A. japonica* harbors relatively higher nucleotide diversities than those of the *A. oxysepala* at both the group and species levels (fig. 1*A*), nucleotide variation patterns of the two species are highly correlated across the nuclear genome (Spearman’s ρ = 0.876) (fig. 1*B* and supplementary fig. S1*A*, Supplementary Material online). The spectrum of allele frequency of the two species was also assessed for the chloroplast and nuclear genome, respectively. For the nuclear genome, both *A. oxysepala* and hybrid exhibit positive Tajima’s *D* values (from 0.139771 to 0.330145) at the group and species levels (table 1). Similarly, while *A. japonica* shows negative value at the species level (Tajima’s *D* = −0.141642), positive Tajima’s *D* values (from 0.081742 to 0.416392) are observed in all the three groups. For the chloroplast genome, both species and their hybrids possess positive Tajima’s *D* values (from 0.134931 to 1.148800) at the group and species levels (table 1). Similar to the genome-wide pattern of nucleotide diversity, the two species also show moderate correlation (Spearman’s ρ = 0.281) for the allele frequency of these 100-kb sliding windows (supplementary fig. S1*B*, Supplementary Material online).

**Table 1:** Nucleotide Diversity (π and *S*) and Allele Frequency (Tajima’s *D*) of *Aquilegia japonica*, *A. oxysepala*, and Their Putative Hybrids at the Group and Species Levels

Species Name^a^	Chloroplast Genome			Nuclear Genome		
	π	*S*^b^	Tajima’s *D*	π	*S*	Tajima’s *D*
*Aquilegia oxysepala*	0.000255	144	0.242280	0.002074	2,370,500	0.139771
Southern	0.000264	135	0.134931	0.002068	2,045,488	0.229483
Northern	0.000242	94	0.742876	0.001907	1,582,564	0.330145
Putative hybrid	0.000261	65	1.148800	0.003626	1,927,265	0.151986
*Aquilegia japonica*	0.000311	174	0.299647	0.002968	3,647,897	−0.141642
Southern	0.000241	99	0.472760	0.002535	2,151,279	0.218674
Middle	0.000202	69	0.677631	0.002434	1,725,215	0.416392
Northern	0.000314	132	0.374022	0.003094	2,700,605	0.081742

^a^Southern, northern, and middle represent all accessions from the southern, northern, and middle group of the two species, respectively.

^b^Only the biallelic variants were considered.

### Phylogenetic Relationship and Genome-Wide Pattern of Genetic Divergence

**Fig. 2.**
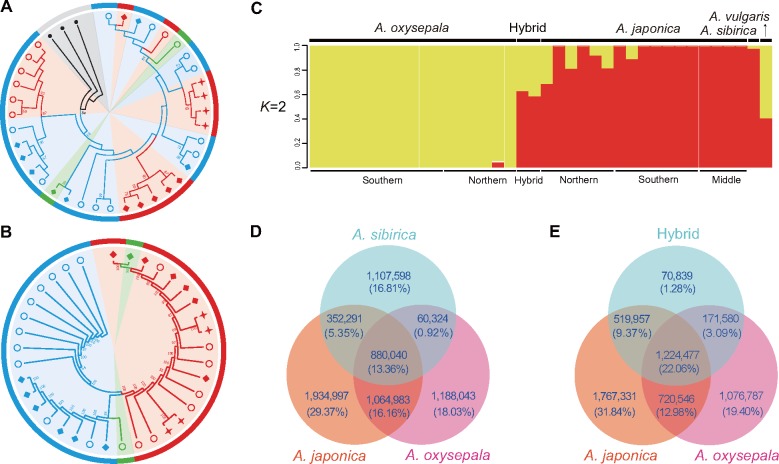
—Phylogenetic and population genetic analyses of the 39 *Aquilegia* accessions used in this study. (*A* and *B*) UPGMA trees of the 39 *Aquilegia* accessions based on chloroplast (*A*) and nuclear (*B*) genomes, receptively. Red, blue, and green colors represent the species *Aquilegia japonica*, *A. oxysepala*, and their putative hybrid, respectively. Black, the three congeneric species *A. sibirica*, *A. vulgaris*, and *A. coerulea*. Circle, diamond, and star indicate the south, north, and middle groups of the two *Aquilegia* species. Length for each branch is not shown. (*C*) Genetic assignments of the *Aquilegia* accessions based on the nuclear genome (*K *=* *2). (*D* and *E*) Venn analyses of the shared and specie-specific among the species *A. oxysepala*, *A. japonica*, their putative hybrid and closely related species *A. sibirica*.

To evaluate the phylogenetic relationships among the 39 *Aquilegia* accessions, ML, NJ, and UPGMA trees were constructed for each chromosome and whole-genome, respectively. All these phylogenetic trees reveal long branch lengths in the three species, *A. coerulea*, *A. vulgaris*, and *A. sibirica* (supplementary figs. S2–S4, Supplementary Material online). In the ML trees, for example, phylogenetic positions of the three congeneric species vary obviously among the seven chromosomes (supplementary fig. S2, Supplementary Material online). In contrast, their topologies in the NJ and UPGMA trees are broadly consistent across the seven chromosomes (supplementary figs. S3 and S4, Supplementary Material online). To this end, we employed the UPGMA method to generate rooted trees in this study. In the chloroplast tree, while most of the clades show low bootstrap support (BS <50%), the three species, *A. coerulea*, *A. vulgaris*, and *A. sibirica*, are placed in the basal clades with high support (BS = 84%) (fig. 2*A* and supplementary fig. S4, Supplementary Material online). Of *A. oxysepala* and *A. japonica*, accessions of the two species and their hybrids are mixed and formed a clade (fig. 2*A* and supplementary fig. S4, Supplementary Material online). In the nuclear UPGMA trees, topologies of the five *Aquilegia* species are highly similar to the chloroplast tree, with the three basal species, *A. sibirica*, *A. coerulea*, and *A. vulgaris*, possessing long branches in both the homozygous and heterozygous trees (supplementary fig. S5, Supplementary Material online) and accessions of *A. japonica* and *A. oxysepala* clustering as a clade (fig. 2*B* and supplementary fig. S4, Supplementary Material online). However, we find that while accessions of the two species fall into two separated lineages at the whole-genome level, they are phylogenetically indistinguishable for chromosomes 3, 4, 5, 6, and 7 (supplementary fig. S4, Supplementary Material online). To further examine the population structures of these *Aquilegia* accessions, we estimated the genetic assignments based on the nuclear genome data set. Estimates of the CV values indicate that the *K *=* *2 is the best genetic cluster (supplementary table S2, Supplementary Material online). Consistent with the above UPGMA topologies, *A. japonica* and *A. oxysepala* show distinct genetic clusters and the two hybrids possess both parental genetic assignments (fig. 2*C*, *K *=* *2). We find that spatial genetic structure is identified when the *K* values are >2 (supplementary fig. S6, Supplementary Material online, *K *=* *3–5), in particular, the two hybrid accessions share the same genetic assignments with geographically closed parental groups. Notably, genetic assignments of *A. sibirica* and *A. vulgaris* are changed in different *K* values, with both species possessing a nuclear genome more similar to *A. japonica*. A similar phenomenon is also found in the Venn analyses, that is, while *A. japonica* and *A. oxysepala* possess the highest proportion of shared variants in the comparisons (29.52–35.04% of the total variants), both *A. sibirica* and putative hybrid share relatively more variants with *A. japonica* (18.71% and 31.42%) compared to *A. oxysepala* (14.28% and 25.15%) at both the chromosome and whole-genome levels (fig. 2*D* and *E*, supplementary tables S3 and S4, Supplementary Material online). However, pair-wise genetic distances between *A. oxysepala* and *A. japonica* (0.003–0.020) are 10 times smaller than those of between the two species and the other three congenic species (0.282–0.358) at each chromosome and the whole-genome levels (supplementary table S5, Supplementary Material online), supporting the above phylogenetic analyses that the two species are sister species. In addition, our comparisons also show that high percentages of species-specific variants are identified in the three species (16.81–29.37% of the total variants) (fig. 2*D* and *E*, supplementary tables S3 and S4, Supplementary Material online).

We next calculated genetic differentiation between *A. japonica* and *A. oxysepala* for the chloroplast and nuclear genomes, respectively. At the whole-genome level, high interspecific genetic divergence is found at both the group (*F*_ST_ = 0.378553–0.509186) and species (*F*_ST_ = 0.400439) levels (table 2). In addition, our results also reveal that the intraspecific *F*_ST_ values between the sympatric groups (*F*_ST_ = 0.378553–0.477948) are not significantly different from those of between the allopatric groups (*F*_ST_ = 0.381113–0.509186) (*t*-test, *P *=* *0.50). In contrast, significantly lower (*t*-test, *P *=* *0.00) intraspecific genetic differentiation (*F*_ST_ = 0.065429–0.135847) is observed compared to those of the interspecific genetic divergence (*F*_ST_ = 0.378553–0.509186) (fig. 1*B* and table 2). In the chloroplast genome, however, no significant differences in the *F*_ST_ values are found in the comparisons at both group (*t*-test, *P *=* *0.59) and species (*t*-test, *P *=* *0.93) levels (table 2). At the chromosome level, our analyses reveal heterogeneous patterns of genetic divergence (Fs, Dxy and *F*_ST_) among the seven chromosomes, with chromosome 4 exhibiting obviously lower genetic divergence compared to the other six chromosomes (fig. 1*B*, supplementary tables S6 and S7, Supplementary Material online). Specifically, 3,430 (87.3%) of the 3,930 fixation sites (Fs) are identified on chromosomes 1 and 2 (supplementary table S7, Supplementary Material online). At the genome-wide level, interspecific *F*_ST_ values of these 100-kb sliding windows are highly similar between the sympatric and allopatric groups of the two species (Spearman’s ρ = 0.83–0.95) (fig. 1*C*). Specifically, the patterns of interspecific genetic divergence between the sympatric/allopatric groups are also highly correlated with that of the species level (Spearman’s ρ = 0.93–0.96) (supplementary fig. S7, Supplementary Material online). These aforementioned comparisons suggest that the two species possess high genetic divergence but nevertheless have similar nucleotide variation patterns in the nuclear genome.

**Table 2:** Genetic Differentiation (*F*_ST_) between *Aquilegia japonica* and *A. oxysepala* at the Group and Species Levels

Comparison Type		Group Name^a^	Chloroplast Genome	Nuclear Genome
Interspecific	Species level	*O*_total_ vs. *J*_total_	0.069224	0.400439
	Sympatric group	*O*_south_ vs. *J*_south_	0.140681	0.477948
		*O*_north_ vs. *J*_north_	0.109502	0.378553
	Allopatric group	*O*_south_ vs. *J*_north_	0.082329	0.381113
		*O*_north_ vs. *J*_south_	0.139866	0.490618
		*O*_south_ vs. *J*_middle_	0.162925	0.488965
		*O*_north_ vs. *J*_middle_	0.211748	0.509186
Intraspecific	Within *A. oxysepala*	*O*_north_ vs. *O*_south_	−0.014809	0.065429
	Within *A. japonica*	*J*_north_ vs. *J*_south_	0.194886	0.135847
		*J*_north_ vs. *J*_middle_	0.096664	0.132636
		*J*_south_ vs. *J*_middle_	0.268048	0.107843

a *O* and *J* represent the species *Aquilegia oxysepala* and *Aquilegia japonica*, respectively; total, accessions of the two species were combined according to their taxonomic rank; south, north, and middle represent all accessions from the southern, northern, and middle groups of the two species, respectively.

### Natural Selection and Demographic History

**Fig. 3.**
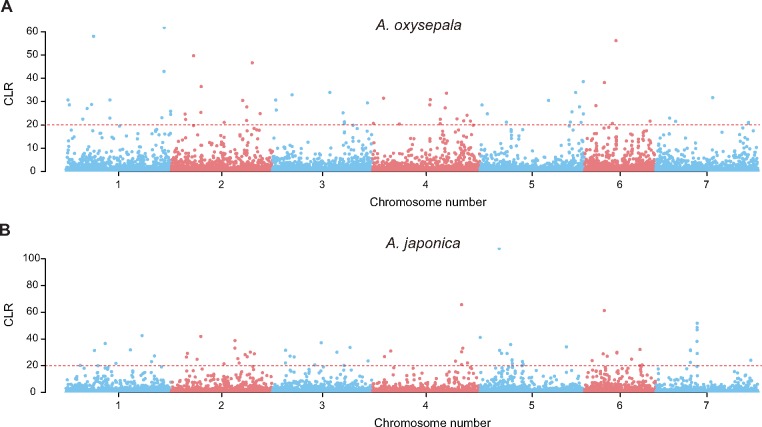
—Genome-wide scanning of the selective sweep across the nuclear genomes of the *Aquilegia oxysepala* and *A. japonica*. Red dashed lines are the CLR value 20 in the two species. Each dot represents one bin on each chromosome.

High genetic divergence between closely related species indicates the possibility of strong natural selection in the process of speciation and adaptation. We therefore identified the selection genomic regions by comparing the HIGD (the top 5% highest) with LND (the top 5% lowest) in the two species. Surprisingly, only one and seven of the 147 species-specific LNDs overlap with the HIGDs (*F*_ST_ >0.613320) in *A. japonica* (π <0.000512) and *A. oxysepala* (π <0.000380), respectively (supplementary fig. S8, Supplementary Material online). More importantly, while 129 (87.8%) of the 147 LNDs share between the two species, only one of these shared LNDs (chr03: 16.8–16.9 Mb) overlaps with the HIGDs (supplementary fig. S8, Supplementary Material online). In contrast, 38 of the shared LNDs (25.9%) exhibit low genetic divergence (the lowest 5%, *F*_ST_ <0.038058) (supplementary fig. S8, Supplementary Material online). We also determined the selective sweeps in each of the two species. Among the 14,000 bins, 66 (0.47%) and 73 (0.52%) selective sweep regions have the CLR values >20 in *A. japonica* and *A. oxysepala* (fig. 3), respectively. Similar to the selection analyses, none of these selection bins overlap between the two species (fig. 3). Demographic histories of *A. japonica* and *A. oxysepala* were also simulated using the nuclear genome data set. Fluctuations in effective population size are found in both species during the past 10^5^ years (supplementary fig. S9*A*, Supplementary Material online). Specifically, the estimated splitting time of the two species is broadly matched with the genetic bottleneck events (supplementary fig. S9*B*, Supplementary Material online). Further simulations of the evolutionary trajectory reveal both historical and recurrent gene flow events among these *Aquilegia* species (supplementary fig. S10, Supplementary Material online). For example, recurrent gene flow is detected from both *A. japonica* and *A. oxysepala* to their putative hybrids. Likewise, historical gene flow is also identified between *A. oxysepala* and other congeneric species.

## Discussion

### Evolutionary Mechanisms Underlying the Rapid Genetic Divergence

Identifying the underlying evolutionary mechanisms that cause genetic divergence between closely related species is key to understanding the evolution of RI. The general view holds that closely related species are expected to show high level of genetic similarity because their recent divergence from a common ancestor, incomplete sorting of ancestral polymorphisms, and gene flow during and after the speciation (Nosil and Feder 2012; Seehausen et al. 2014; Wolf and Ellegren 2017). In the case of Darwin’s finches, for example, polyphyletic relationships with high genetic similarities were found among the morphologically and ecologically distinct species (Lamichhaney et al. 2015, 2018). Population genomic analyses have demonstrated that the combination of ancient polymorphisms, gene flow, and natural selection has resulted in a heterogeneous pattern of genetic divergence among these finch species (Han et al. 2017). In plants, a similar phenomenon was observed in wild sunflowers in which genomic divergences between the sympatric and parapatric species pairs are shaped by natural selection, local genomic landscape, and recombination rate (Renaut et al. 2013, 2014).

In this study, population genomic analyses have confirmed our previous observation that the two species, *A. oxysepala* and *A. japonica*, are phylogenetically indistinguishable at the chloroplast genome level (Li et al. 2014). It is not surprising that rapid adaptive radiation together with low mutation rate of the chloroplast genome has resulted in the low phylogenetic resolution among the columbine species (Fior et al. 2013), particularly between the two closely related species that are diverged recently (<50,000 years) with ongoing gene flow (Li et al. 2014). With this reasoning, the shared ancestral polymorphisms as well as gene flow might be the potential evolutionary mechanisms that generated the admixture genetic pattern of the two species at the chloroplast genome. In fact, low genetic divergence at the nuclear genome was also observed in the North American and European columbine species (Hodges and Arnold 1994; Whittall et al. 2006; Cooper et al. 2010; Noutsos et al. 2014; Garrido et al. 2017). For example, while the two species, *A. formosa* and *A. pubescens*, are morphologically and ecologically distinct, they are indistinguishable at the nuclear genes and interspecific hybridization occurs frequently in the contact zone (Hodges and Arnold 1994; Whittall et al. 2006; Cooper et al. 2010; Noutsos et al. 2014). Similar admixture genetic pattern was also found in the Iberian columbine species complex where all sampled populations are clustered as geographically structured lineages instead of their taxonomic ranks (Garrido et al. 2017). Nevertheless, both this and our previous study (Li et al. 2014) revealed high genetic divergence between *A. oxysepala* and *A. japonica* in the nuclear genome. Importantly, genome-wide comparisons of the sympatric and allopatric groups between the two species have revealed highly similar nucleotide variation patterns (*F*_ST_ and π) across the nuclear genome. Based on the comparative genomic analyses of 10 worldwide columbine species, Filiault *et al.* (2018) proposed a speciation with hybridization model, with *A. japonica* sharing the MRCA with *A. sibirica* but experiencing recent hybridization with the basal species *A. oxysepala*. Under this hypothesis, the observed high genetic divergence is possibly due to the distant phylogenetic relationships between *A. japonica* and *A. oxysepala*. However, this speciation model mainly relied on the nuclear genome with only one accession for each species and without taking into account of the chloroplast genome. Consistent with previous studies (Bastida et al. 2010; Fior et al. 2013; Li et al. 2014), our chloroplast and nuclear phylogenies clearly confirmed the sister group relationship between *A. oxysepala* and *A. japonica* at the population level. According to the Flora of China and our observations, *A. japonica* and *A. oxysepala* are sympatrically distributed in the northeastern China but show obvious differences in ecological niches and morphological traits. In contrast, the allopatric species pair, *A. japonica* and *A. sibirica* (distributed in northwestern China and Central Asia), share highly similar ecological habitats and morphological traits. Previous phylogenetic and biogeographic analyses based on the chloroplast genome suggested a recolonization origin of these Asian columbine species from European stock (Fior et al. 2013). Taking these attributes together, we propose a possible scenario that *A. sibirica* might have split from the common ancestor at first; then, adaptive speciation occurred between *A. japonica* and *A. oxysepala* during the recolonization process.

Given that divergence between *A. japonica* and *A. oxysepala* is broadly matched with the shrinkage of effective population size and the nucleotide variation patterns (*F*_ST_ and π) are highly similar at both the group and species levels, we propose that the observed high genetic divergence in the nuclear genome is at least partially due to the geographical isolation at the initial speciation stage. In fact, this hypothesis was documented in the *Heliconius* spp. butterflies in which genomic divergences are obviously higher in the species pairs with geographical isolation at the early speciation stage compared to those without initial geographical isolation (Martin et al. 2013). On the other hand, it was found in the paraphyletic species pair, *Populus euphratica* and *P. pruinosa*, that divergent sorting of ancient polymorphisms followed by genome hitchhiking has driven the genome-wide genetic divergence (Ma et al. 2018). In our case, high proportion of species-specific variants together with very few shared selective candidates (e.g., selective sweeps) suggests that divergent sorting of ancestral polymorphisms in the ancestral allopatric populations and divergence hitchhiking during and after the speciation might have further promoted the genetic divergence between the two species. Taken together, our population genomic analyses based on both chloroplast and nuclear genomes provide a framework on how the combination of shared ancestral polymorphisms, allopatric isolation, and natural selection have shaped the genomic architectures of the two ecologically isolated species.

### Speciation with Allopatric Isolation Followed by Adaptation to Contrasting Environments

The question of whether the reproductive barrier between incipient species evolves with or without gene flow has long been a central topic in the evolutionary biology (Feder et al. 2013; Wolf and Ellegren 2017). In the geographic (allopatric) model, speciation with gene flow should be very rare in nature as it will break down the reproductive barriers by homogenizing the gene pools (Mayr 1942; Oka 1957; Orr 1995). However, Wu (2001) argued that gene flow can occur at genomic regions that are selectively neutral during the speciation. Examples of the genic speciation model were typically observed in the *Heliconius melpomene* where the RIs between different races evolve rapidly in the face of gene flow excepting for genomic regions associated with the adaptive aposematic colors (Dasmahapatra et al. 2012; Nadeau et al. 2012). In our study, while *A. oxysepala* and *A. japonica* diverged from each other very recently, a suite of adaptive traits (e.g., plant height and leaf area) that are specialized to contrasting ecological niches have been developed (Li et al. 2011, 2014). In particular, demographic simulation reveals gene flow in both the chloroplast (Li et al. 2014) and nuclear (this study) genomes. These findings point to possible ecological speciation of the two species. With this scenario, one would expect to see a heterogeneous pattern of genetic divergence between the incipient species, with natural selection generating the genomic islands of divergence but gene flow homogenizing the rest of the genome (Via and West 2008; Nosil et al. 2009). However, our study reveals unexpectedly high genome-wide genetic divergence between *A. oxysepala* and *A. japonica*. The heterogeneous patterns of genetic divergence among the chromosomes are mainly due to the unique evolutionary history of chromosome 4 (Filiault et al. 2018). With this reasoning, it is more likely that allopatric isolation precludes genetic exchanges at the initial speciation stage. Gene flow detected between the two species possibly occurred after the speciation and/or during the adaptation process. The putative hybrids of the two species were formed through hybridization after secondary contact. The allopatric divergence with secondary contact model was also reported in the north American lake whitefish in which repeated divergence of benthic normal and limnetic dwarf ecotypes from allopatric populations followed by secondary contact during the LGM has led to the establishment of RIs between different whitefish ecotypes (Rogers and Bernatchez 2007; Renaut et al. 2011; Gagnaire et al. 2013). To further test this speciation with allopatric isolation model, we assessed the impacts of natural selection on RI evolution. In the case where natural selection acts on a large number of small effect genes during the speciation, one would expect to identify candidate genomic regions that show differentially high genetic divergence between *A. oxysepala* and *A. japonica* with low nucleotide diversity within both species. However, very few candidate genomic regions of this kind are identified in the nuclear genome of the two species. In contrast, candidate selective sweep regions differ almost entirely between the two species. In this scenario, adaptive selection in the process of ecological specialization, rather than divergent selection during speciation, associated with allopatric isolation cause the observed high genetic divergence between the two species. Another possibility is that divergent selection acts on few large effect genes in the process of speciation; then, allopatric isolation during the ecological specialization further promotes the genetic divergence. Taken together, these observations above suggest that speciation between the two ecologically and morphologically distinct species has been involved in an initial stage of allopatric isolation without gene flow, and then, natural selection further promotes the genetic divergence. Our study provides a genome-wide view of how RIs evolve between closely related *Aquilegia* species.

## Supplementary Material

Supplementary data are available at *Genome Biology and Evolution* online.

## Supplementary Material

Supplementary Data

## Notes

**Data deposition:** All whole genome sequences generated in this study were deposited to GenBank under the project number PRJNA433751.
