# Supplementary figures and images for: Rapid Divergence Followed by Adaptation to Contrasting Ecological Niches of Two Closely Related Columbine Species Aquilegia japonica and A. oxysepala
Source: Genome Biol Evol. 2019 Feb 21;11(3):919–30. doi: 10.1093/gbe/evz038 (PMC6433176; doi:10.1093/gbe/evz038)

A

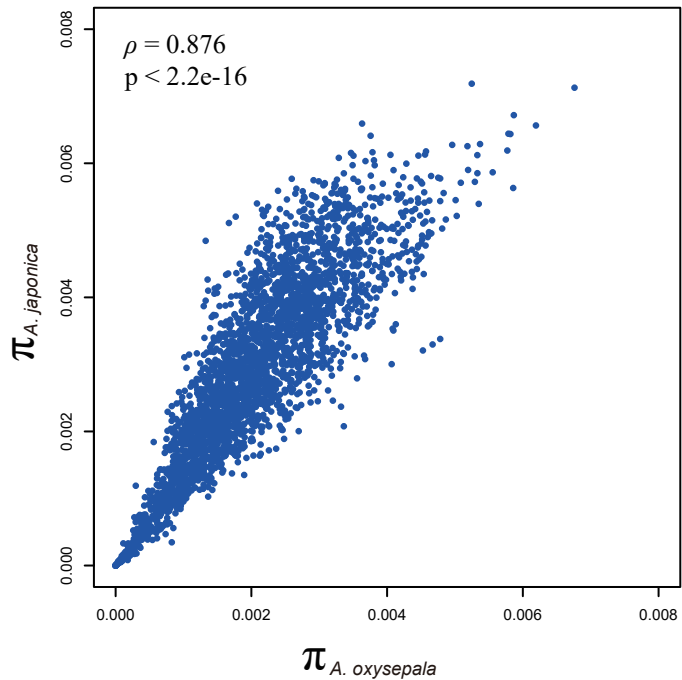

B

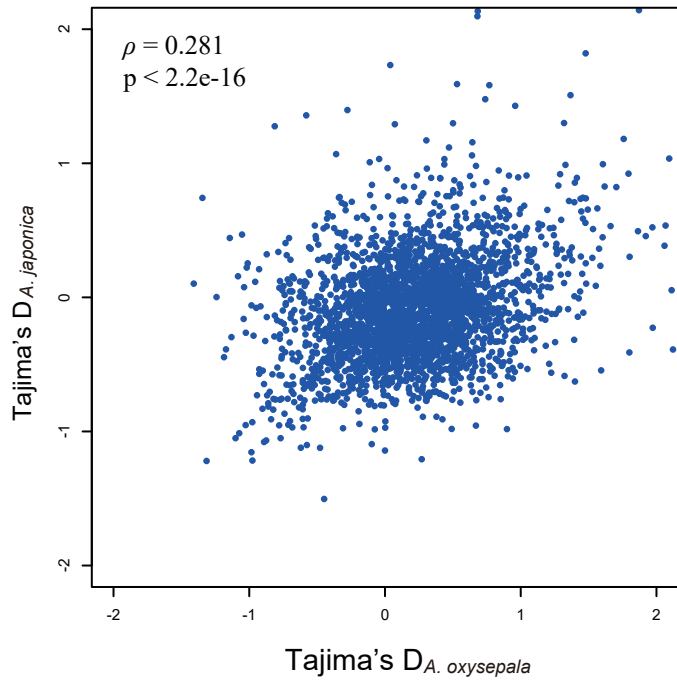

Supplement: Supplementary Data [file evz038_supp.zip › Fig. S1.pdf]

A

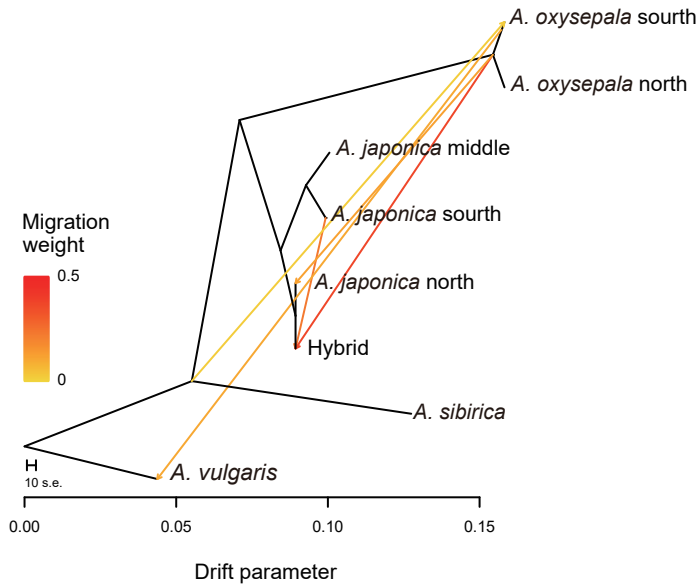

B

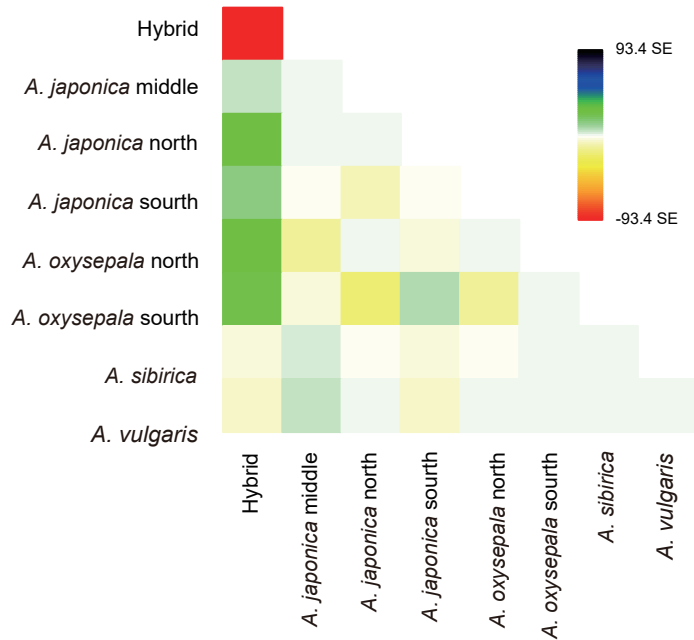

Supplement: Supplementary Data [file evz038_supp.zip › Fig. S10.pdf]

Chr1

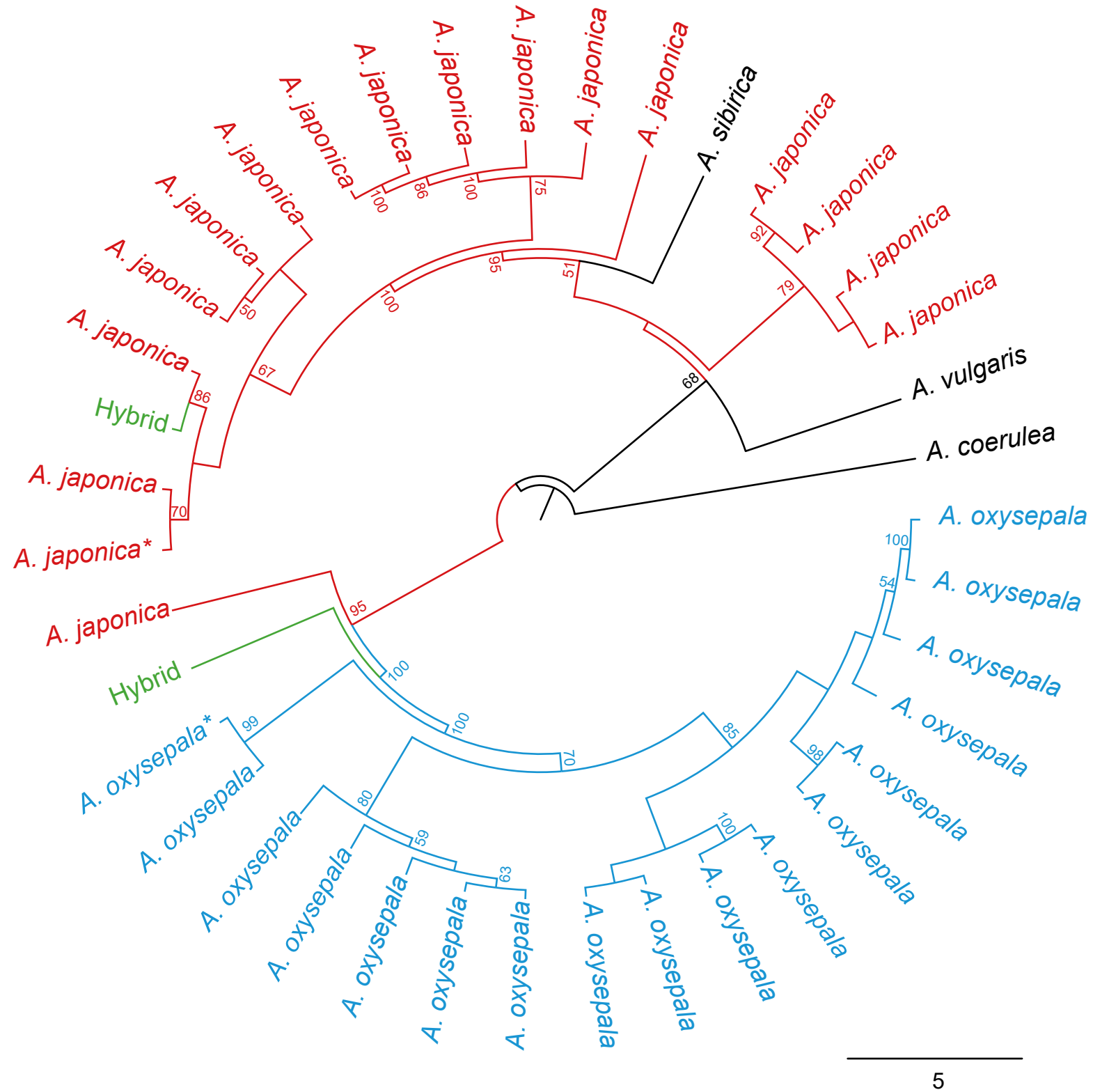

Chr2

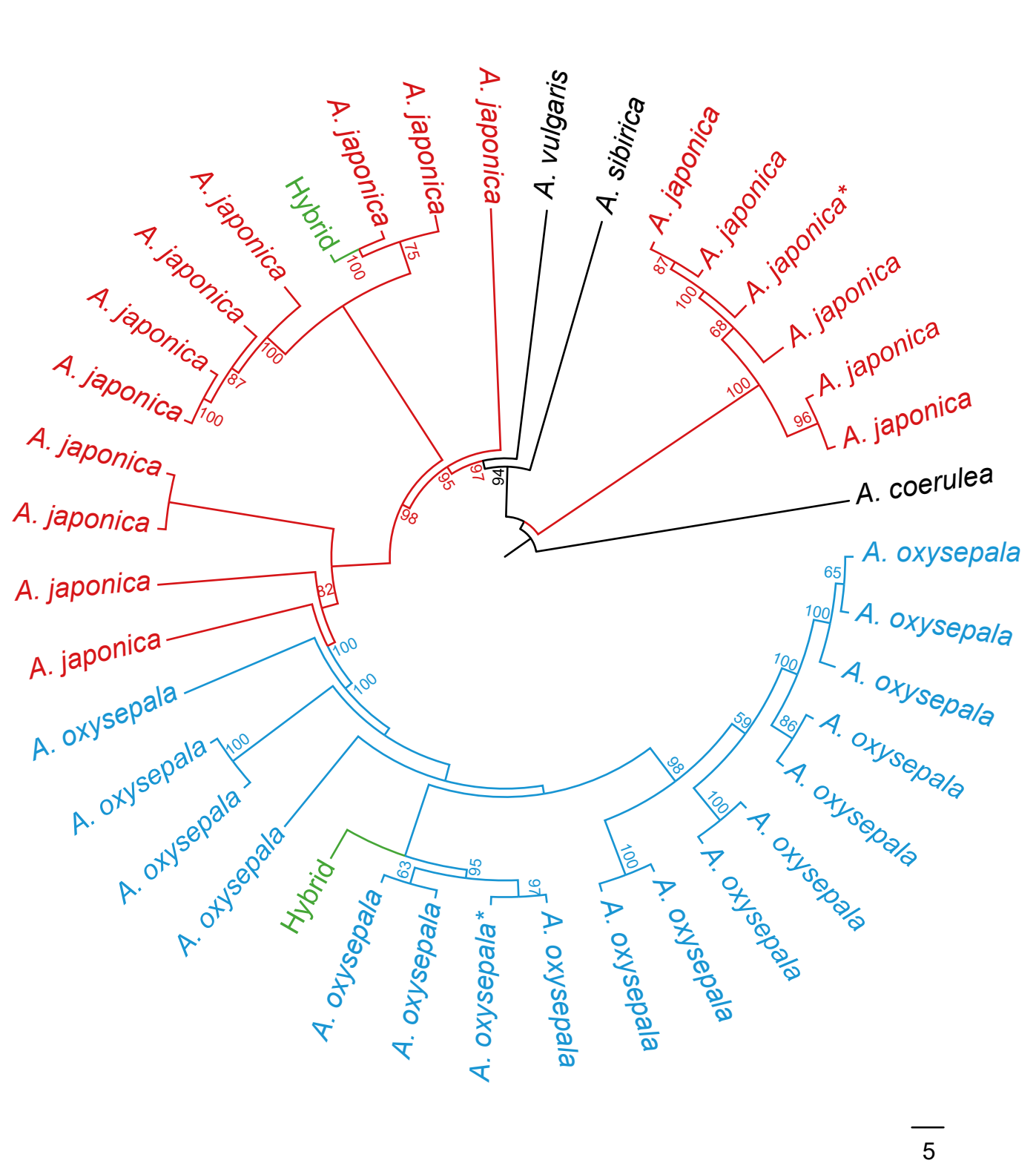

Chr3

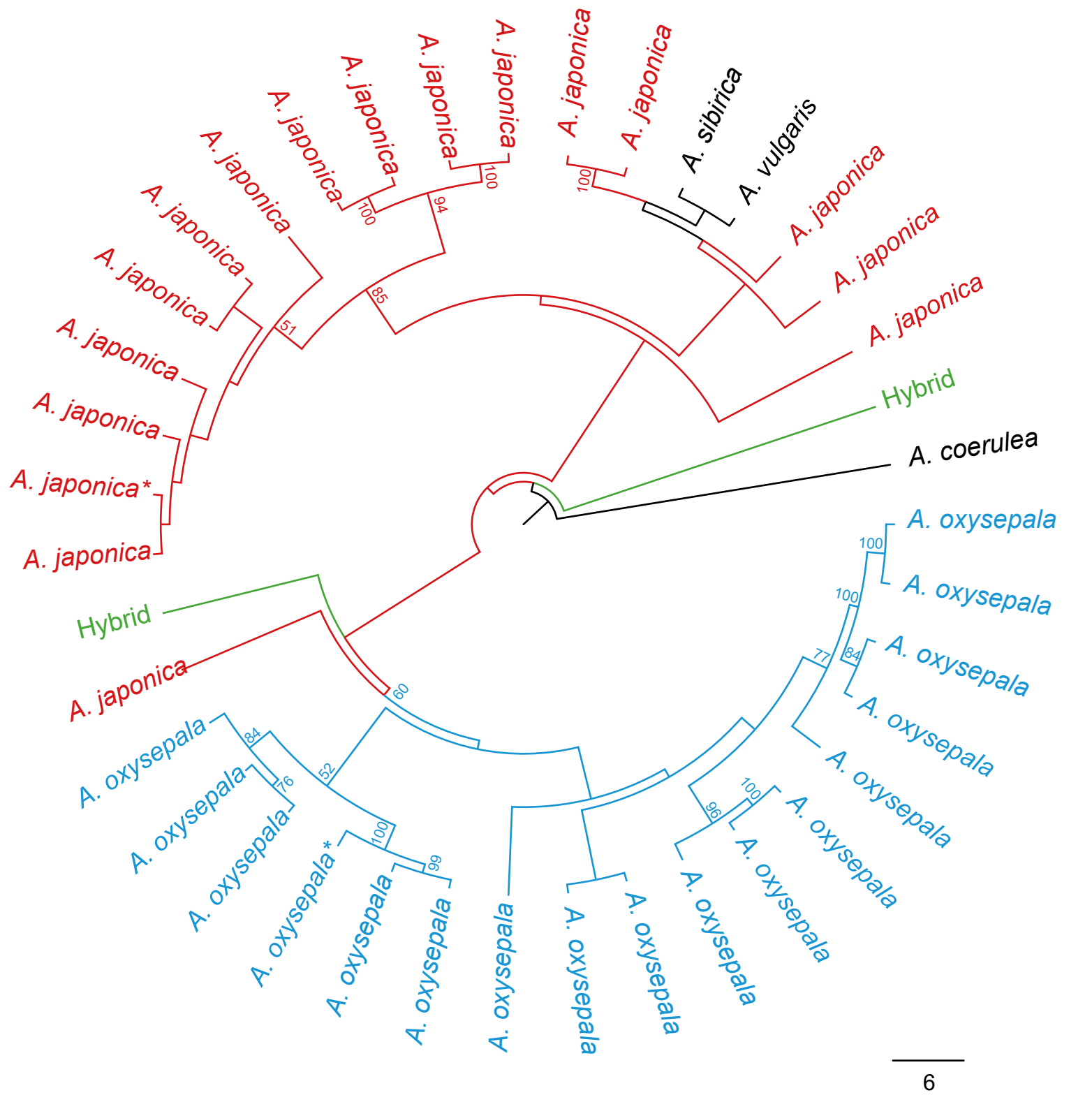

Chr4

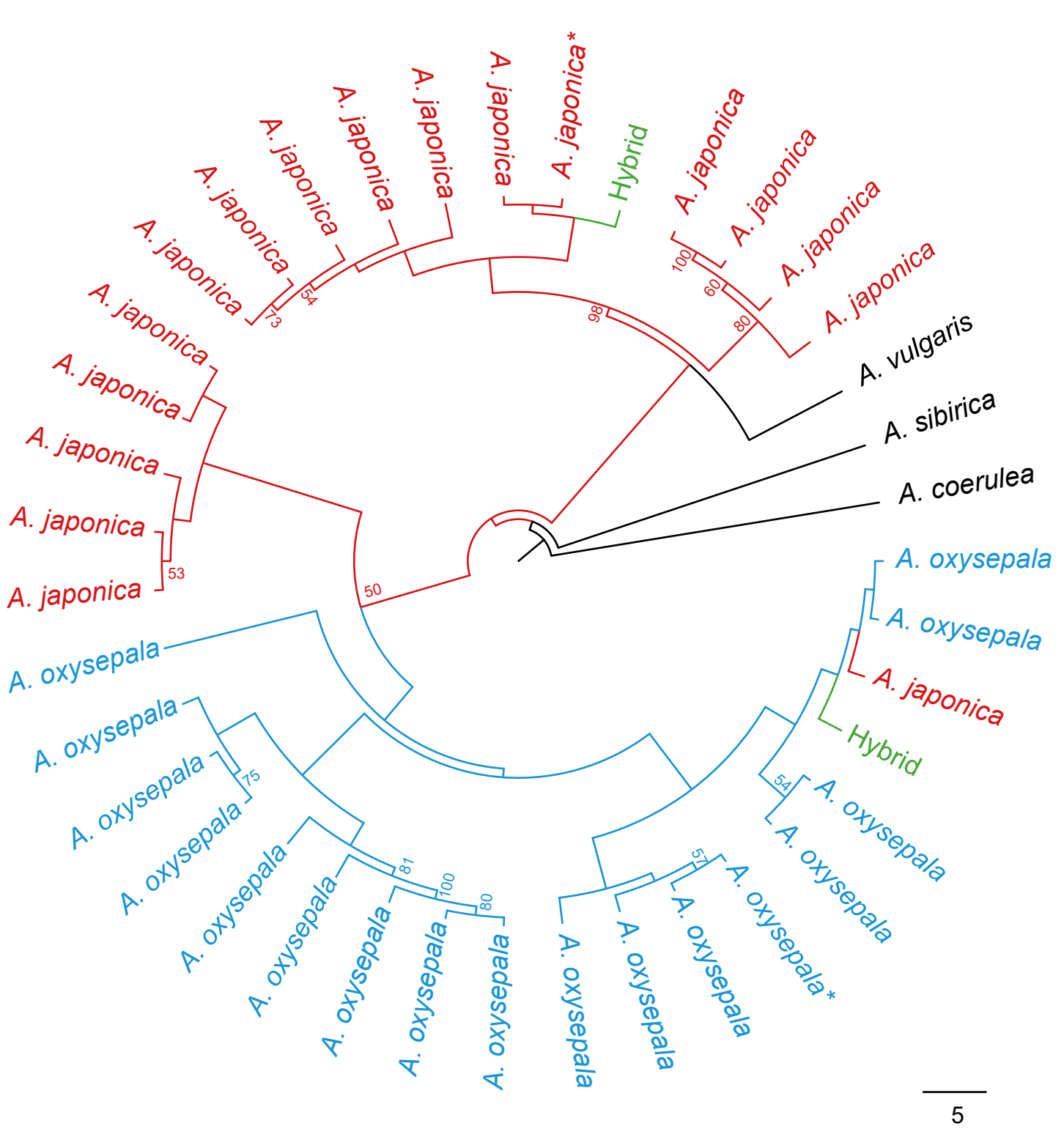

Chr5

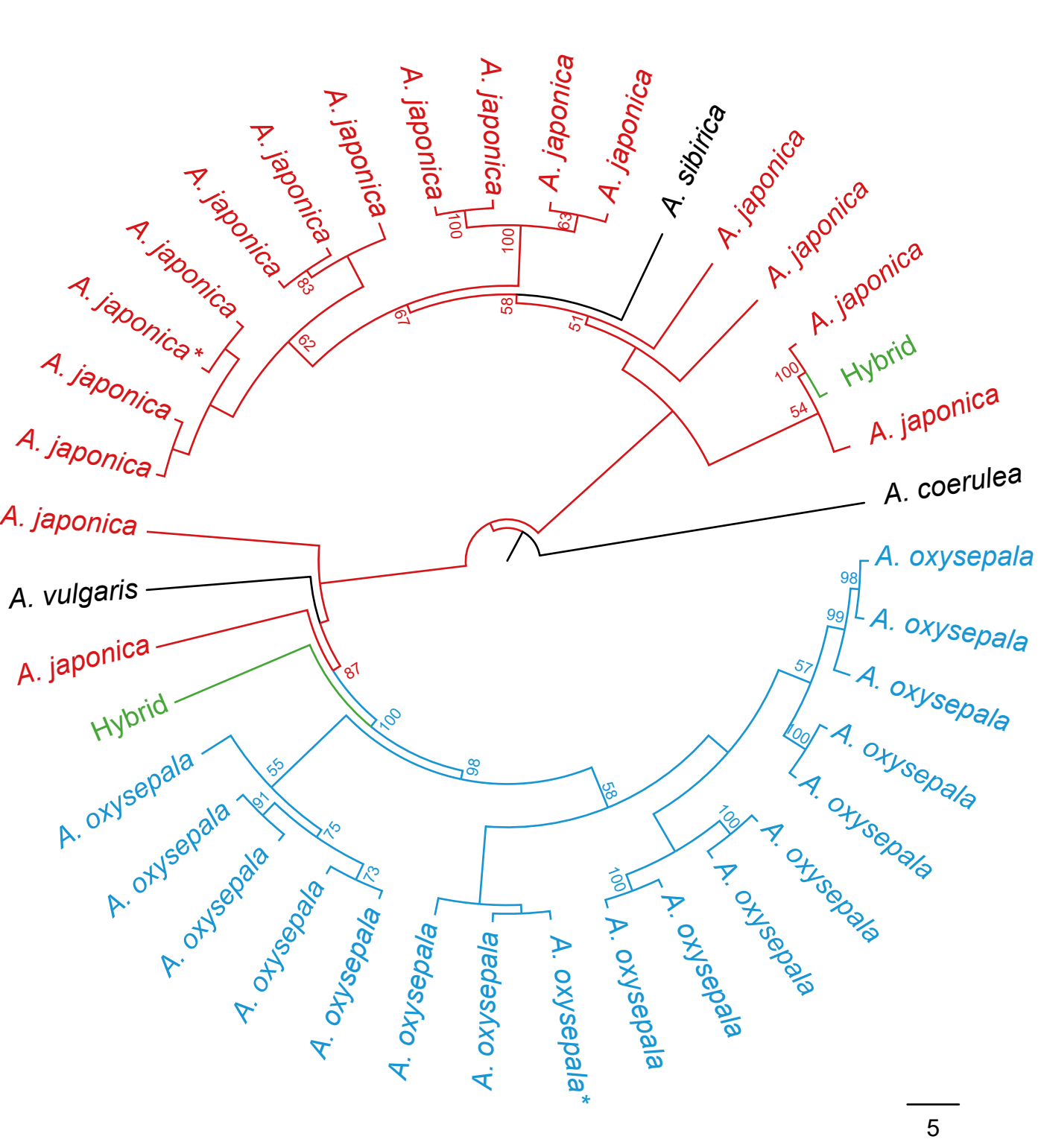

Chr6

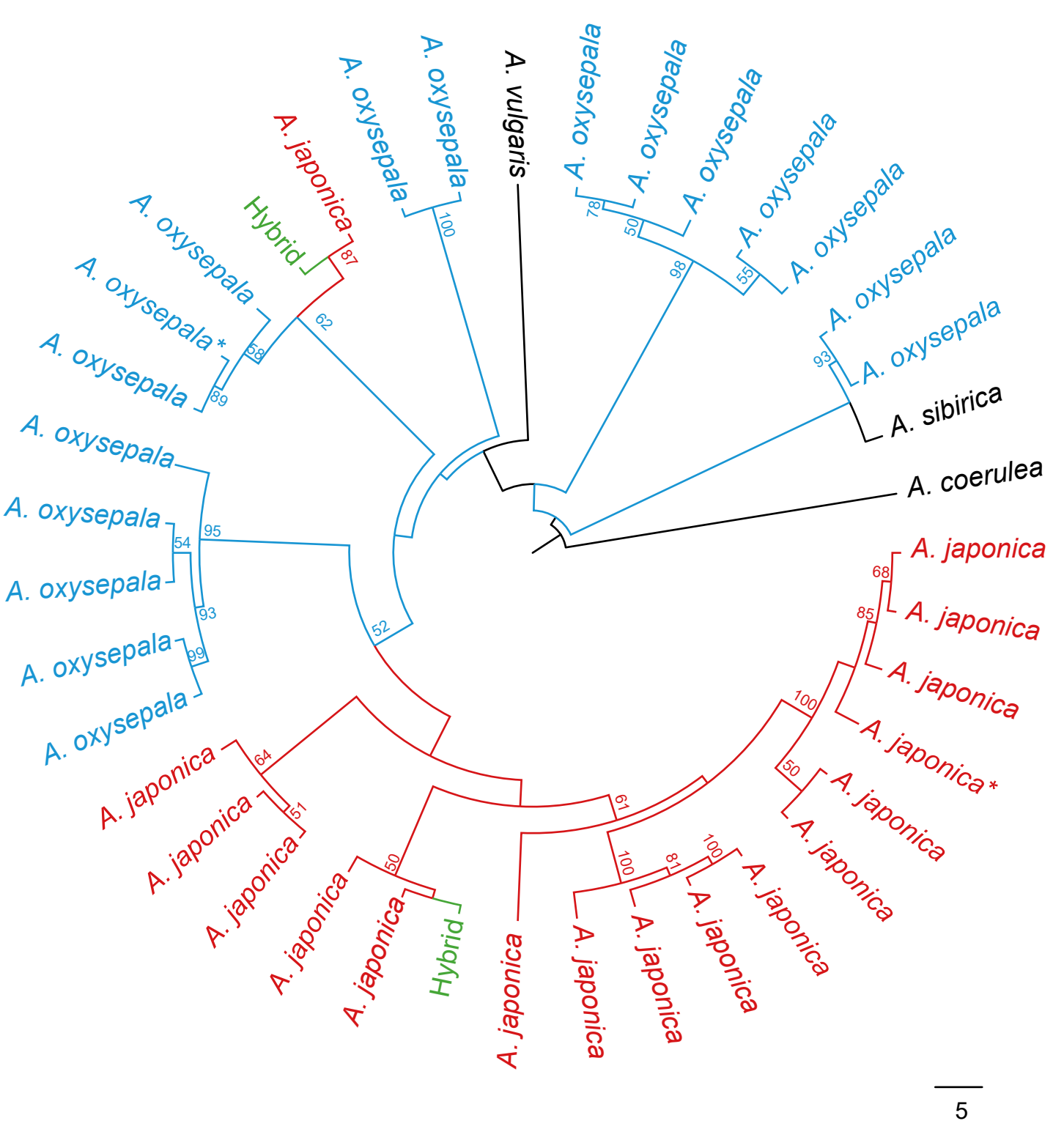

Chr7

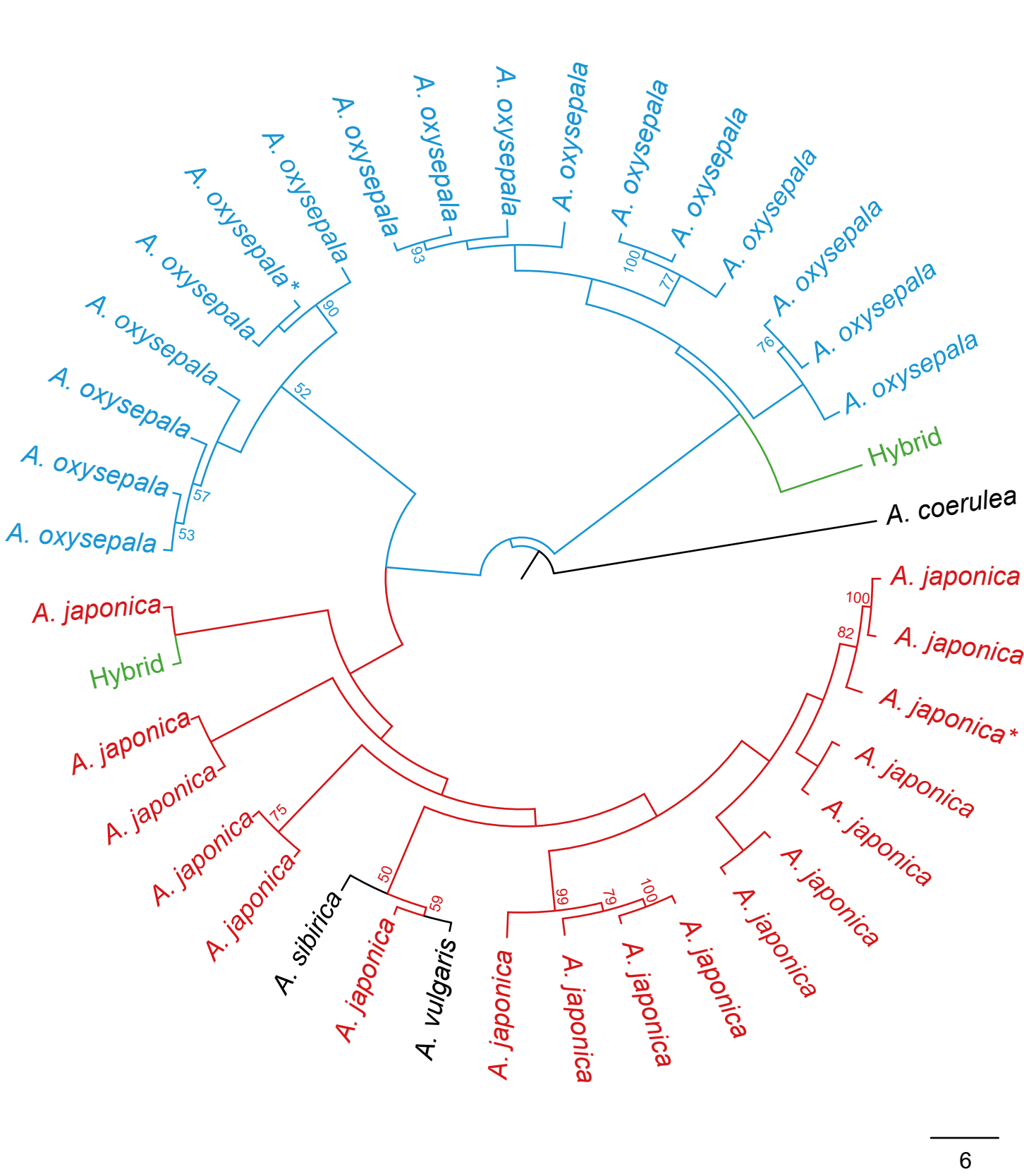

All

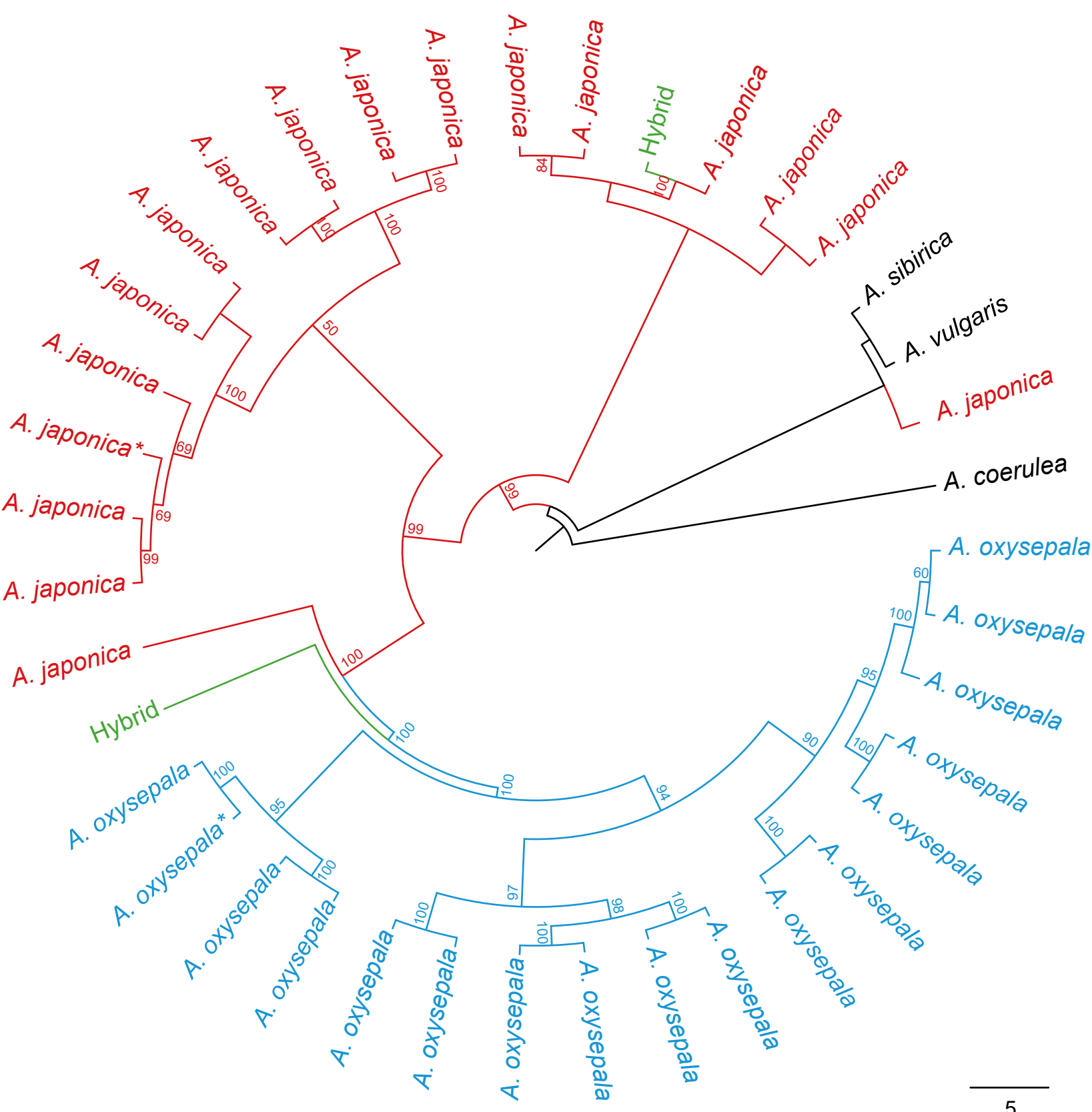

Chloroplast

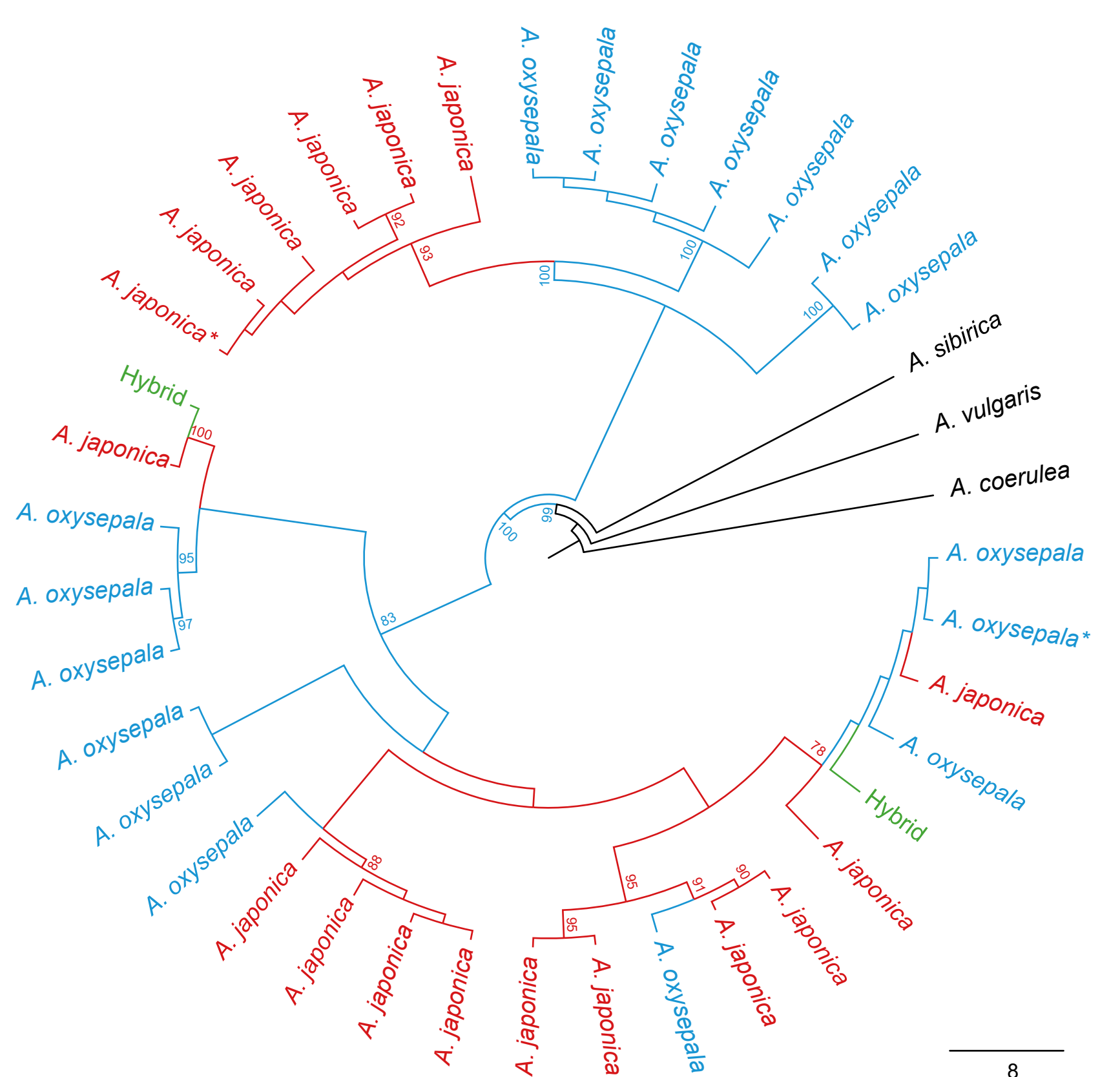

Supplement: Supplementary Data [file evz038_supp.zip › Fig. S2.pdf]

Chr1

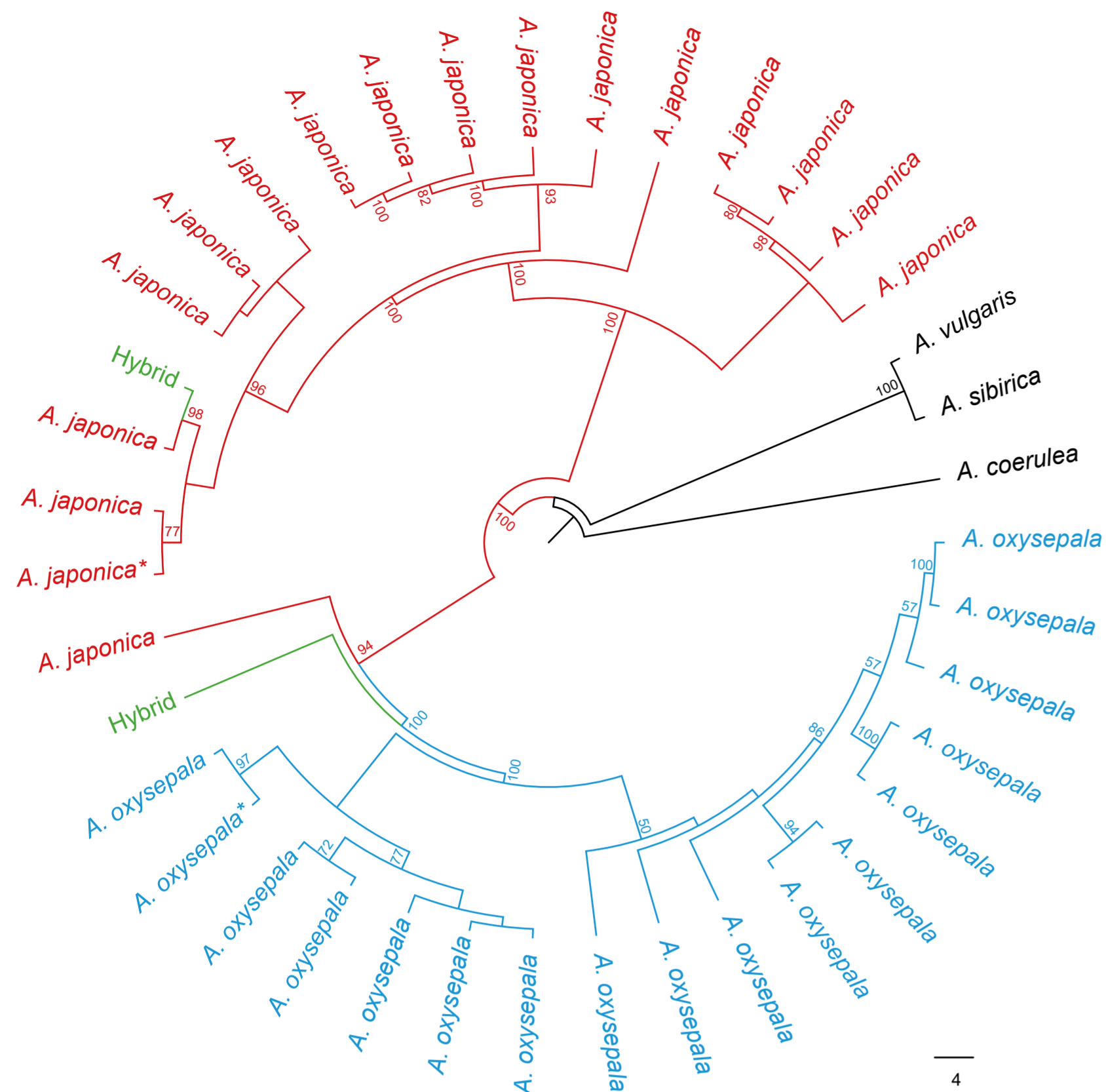

Chr2

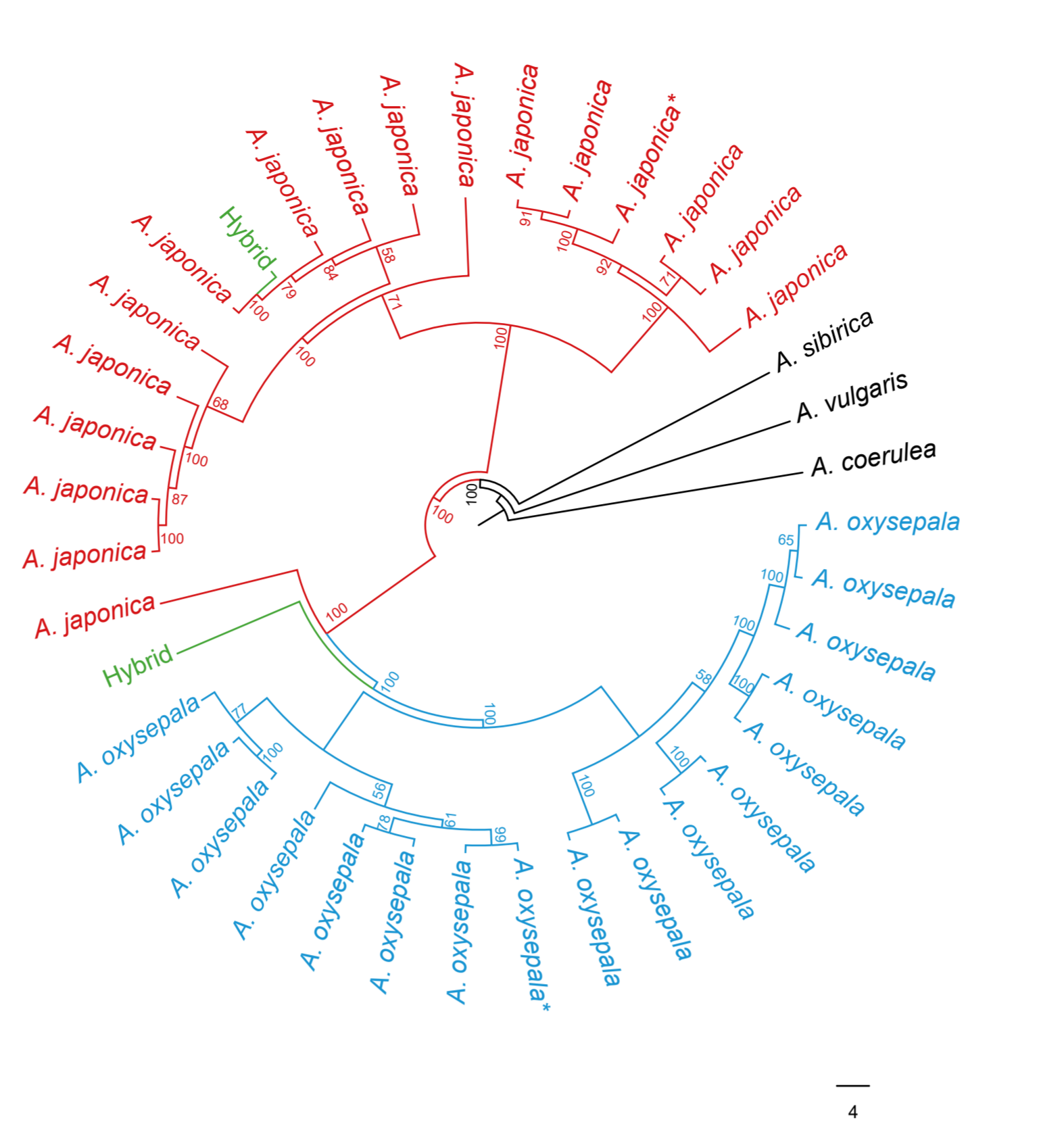

Chr3

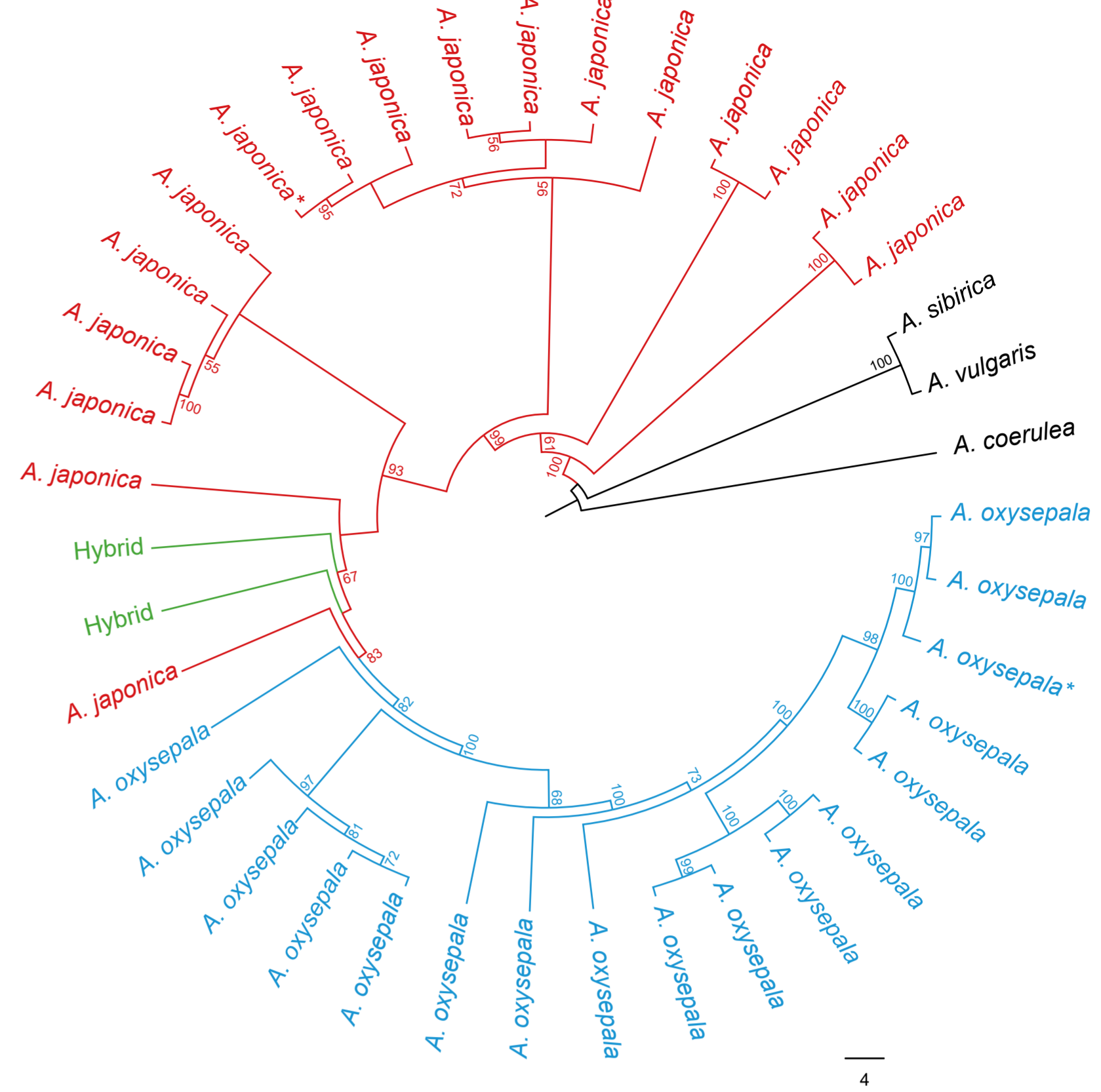

Chr4

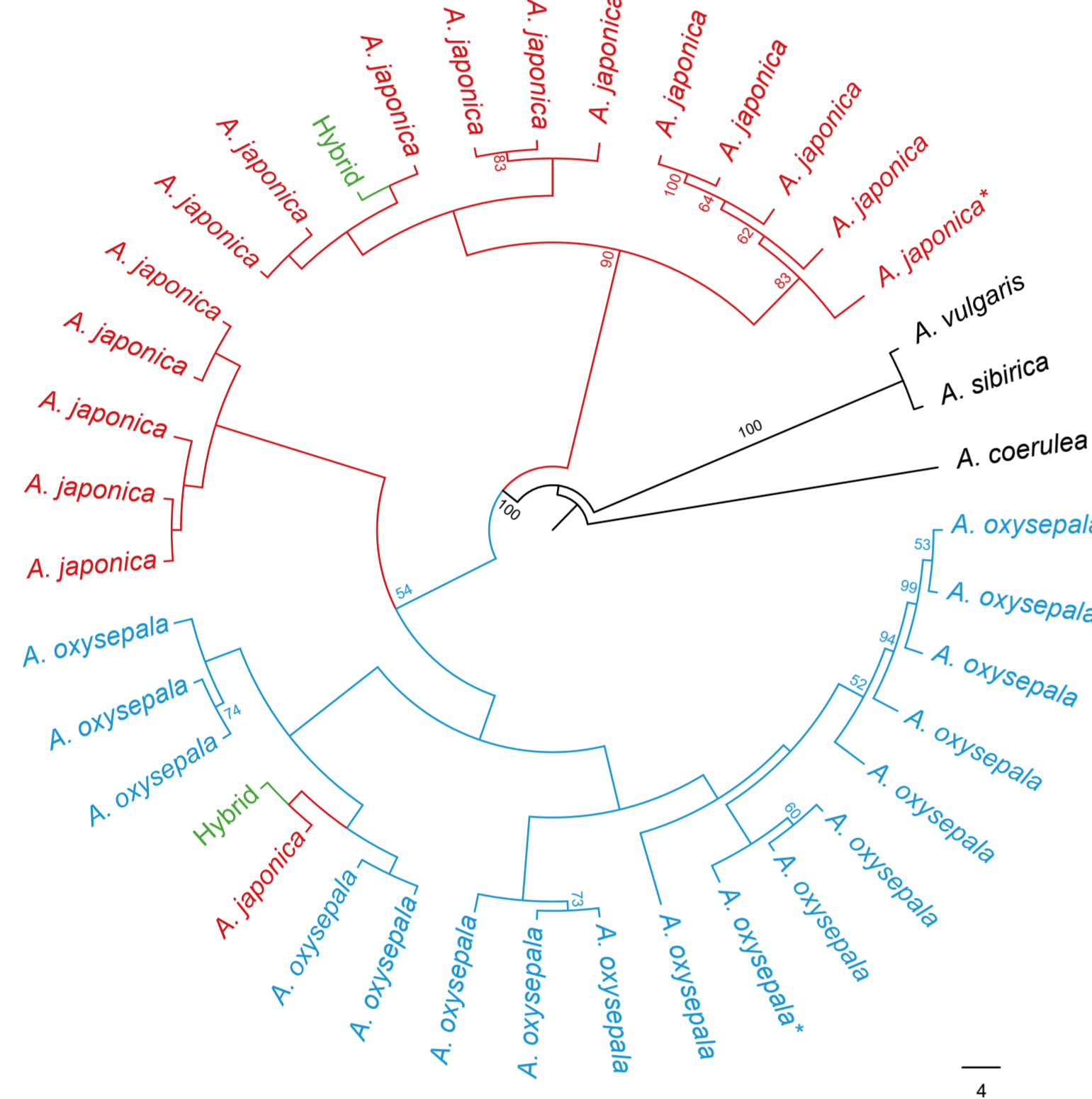

Chr5

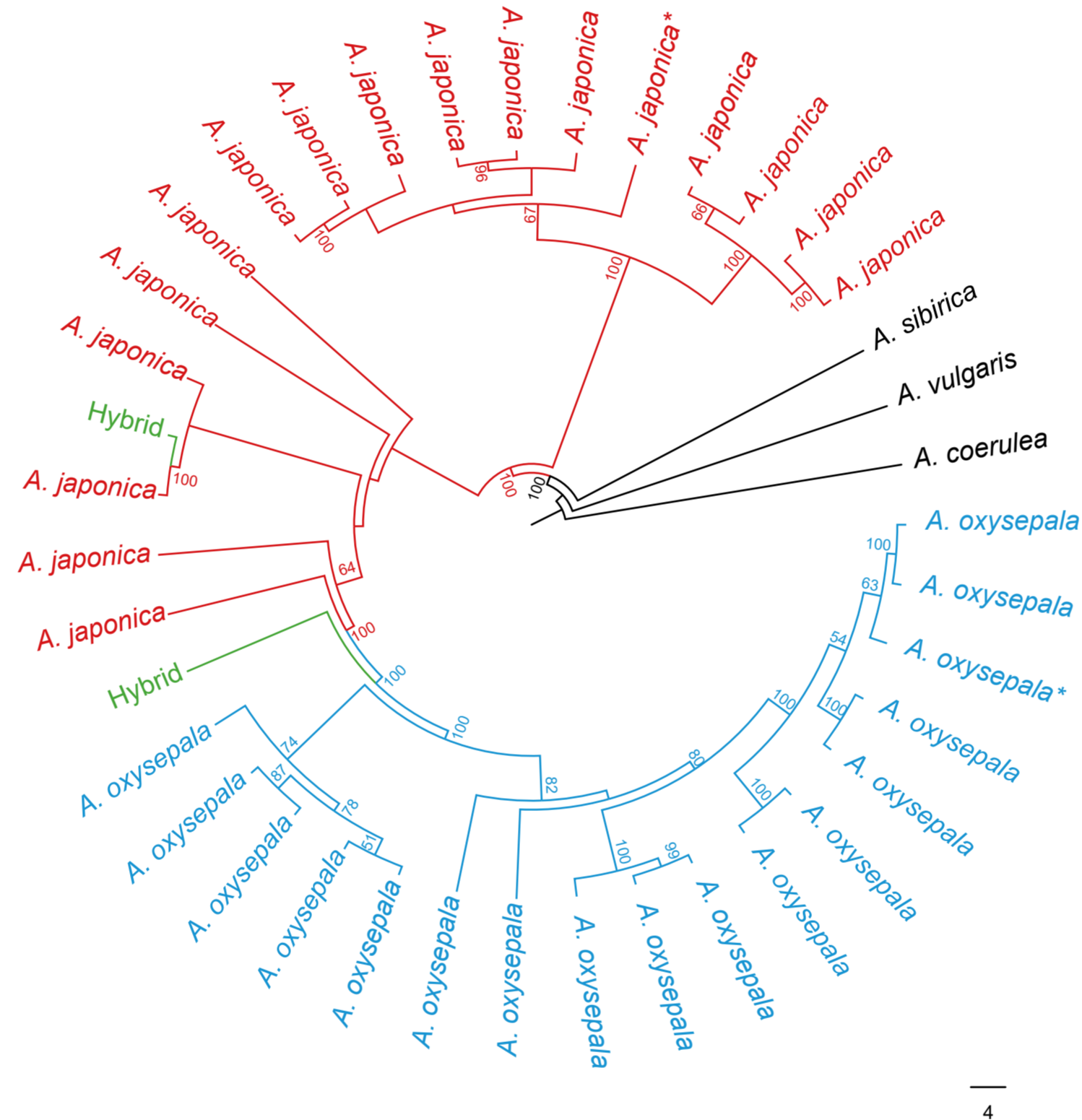

Chr6

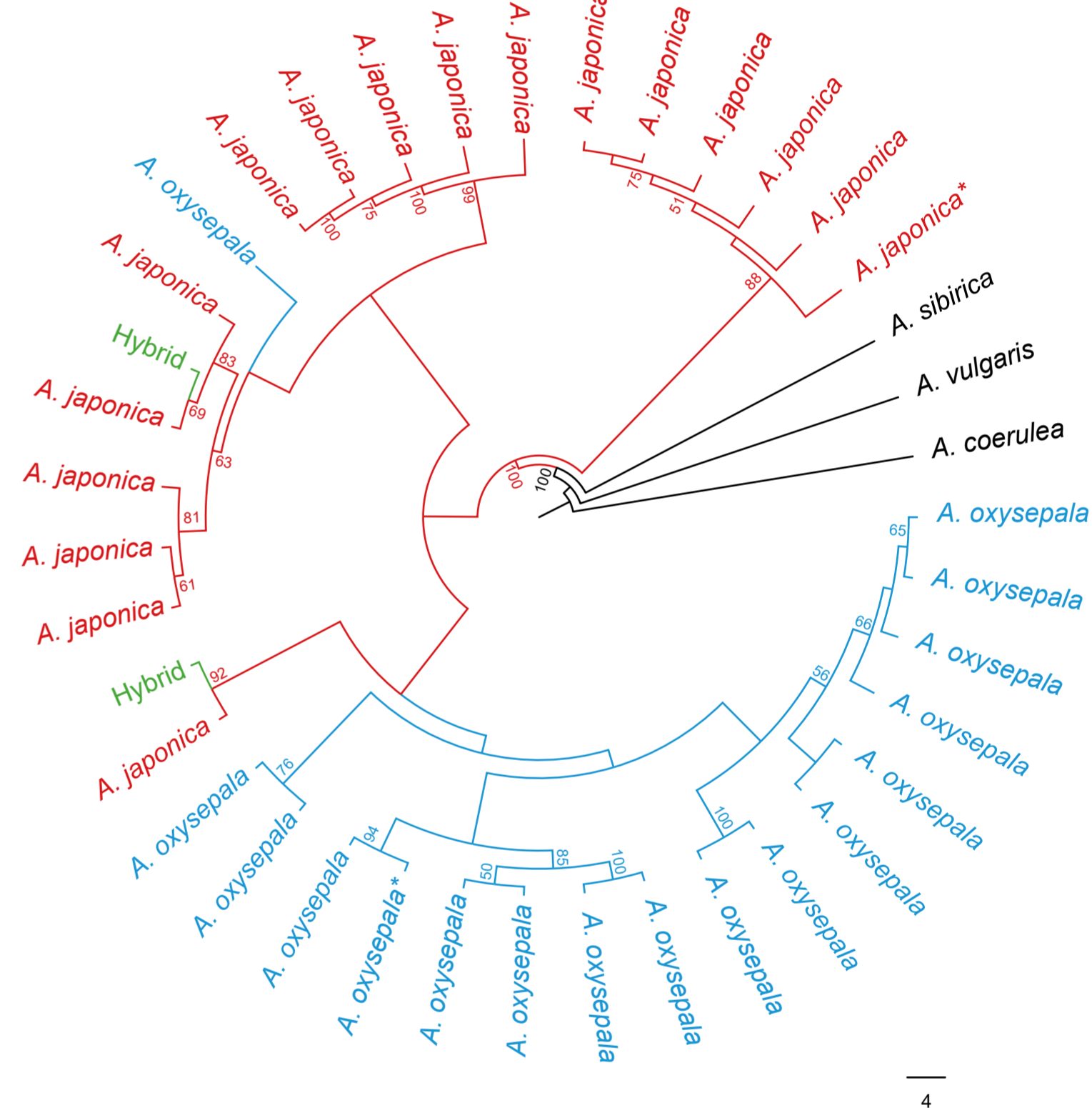

Chr7

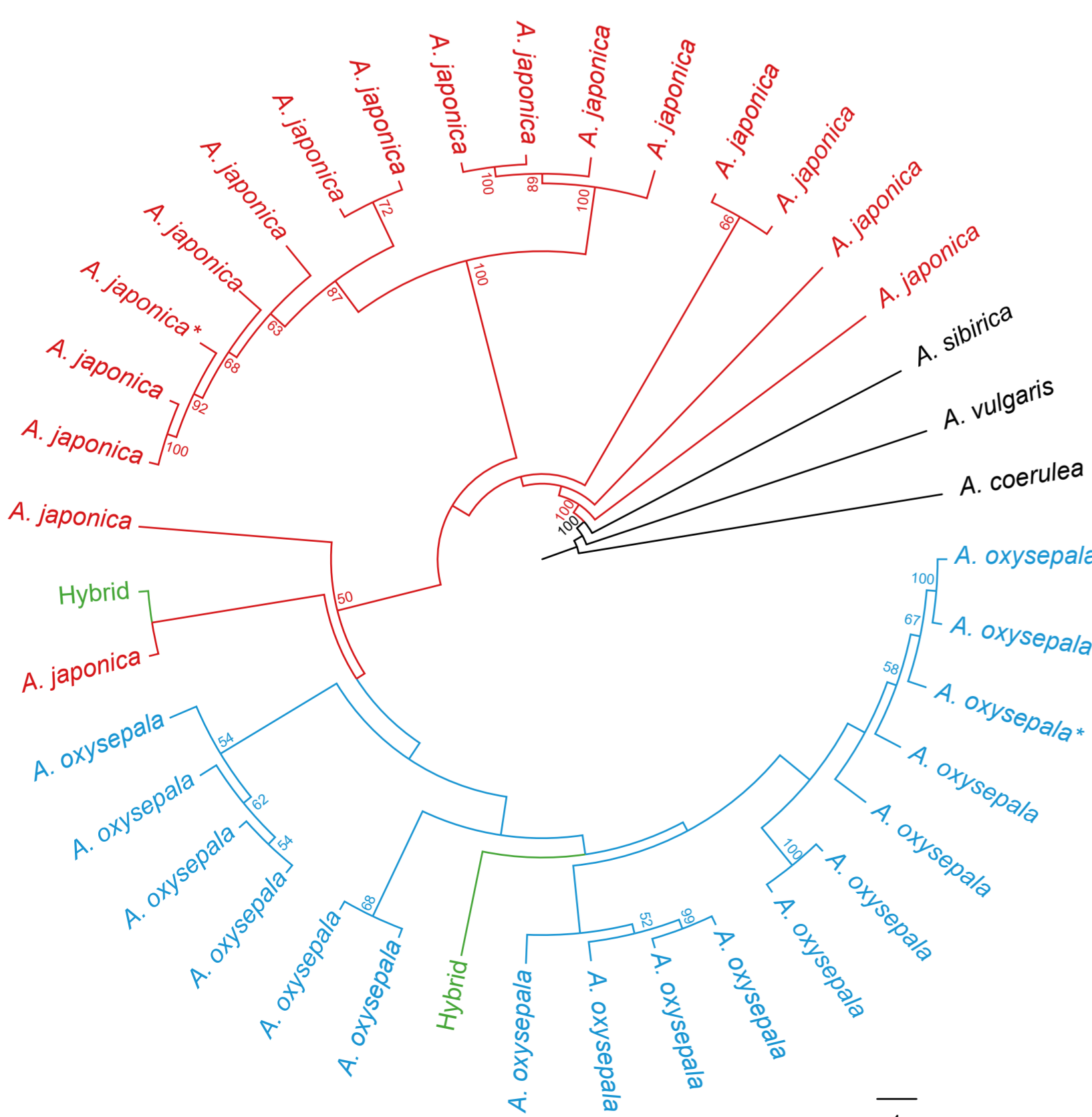

All

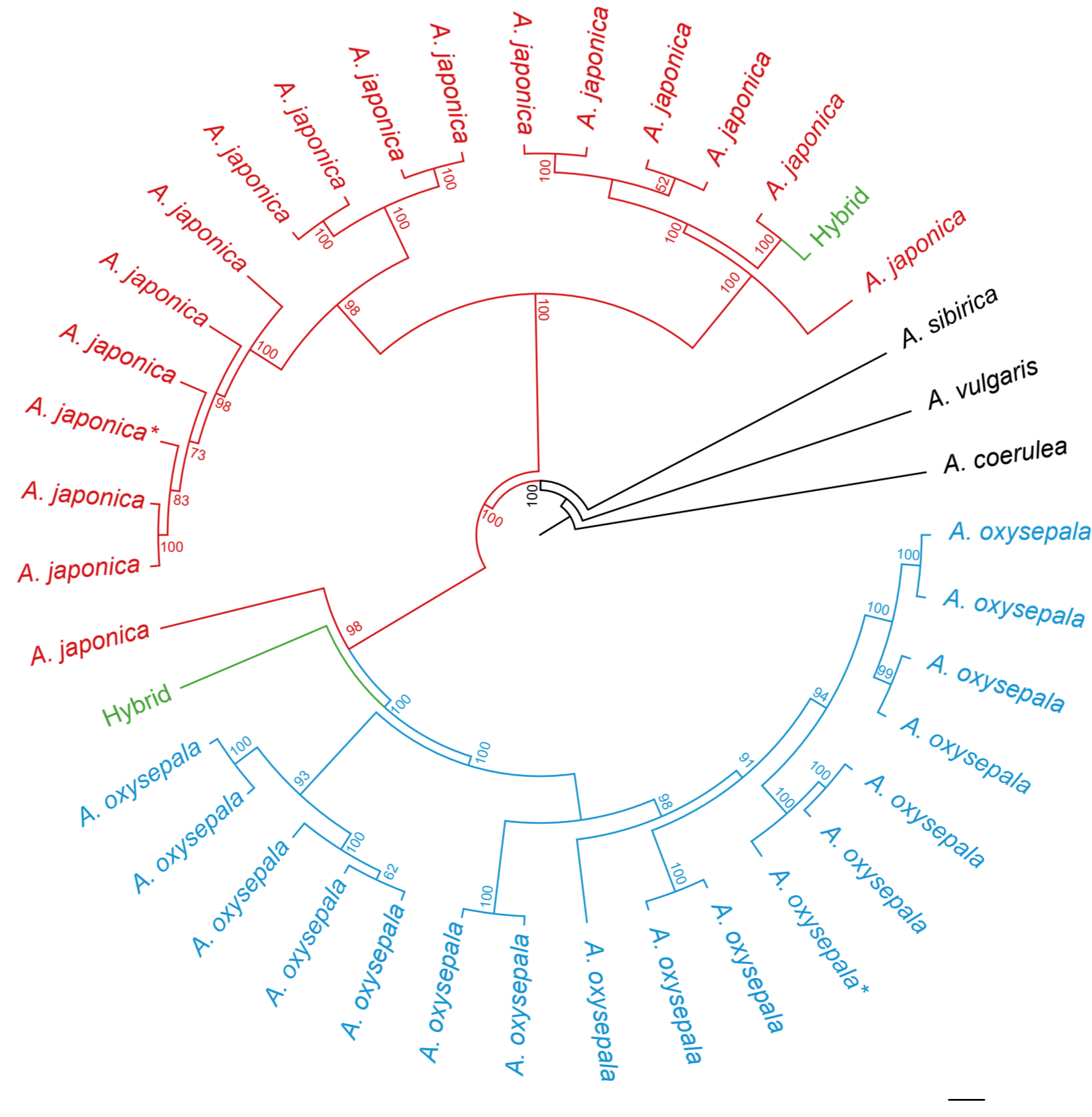

Chloroplast

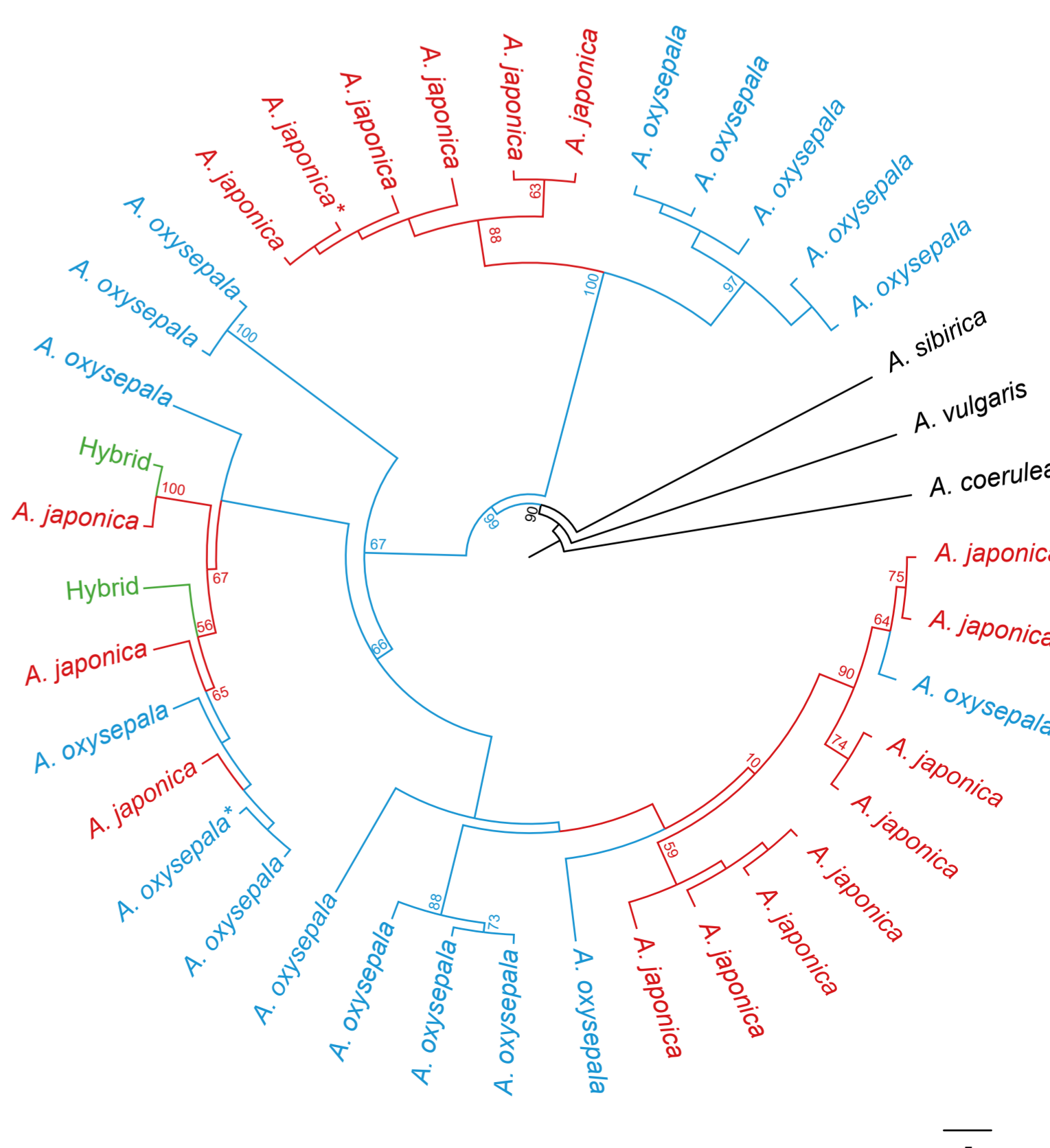

Supplement: Supplementary Data [file evz038_supp.zip › Fig. S3.pdf]

Chr1

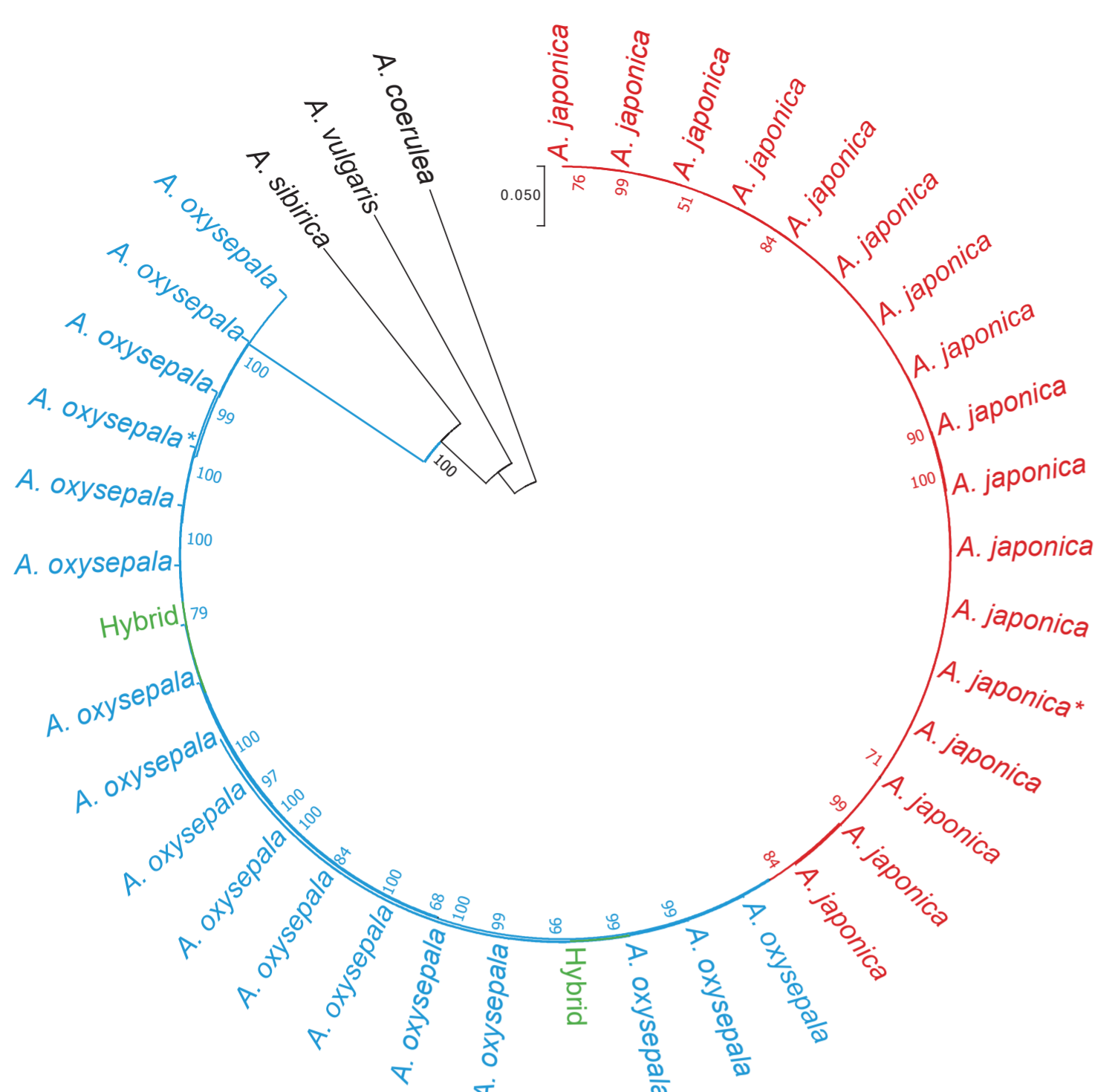

Chr2

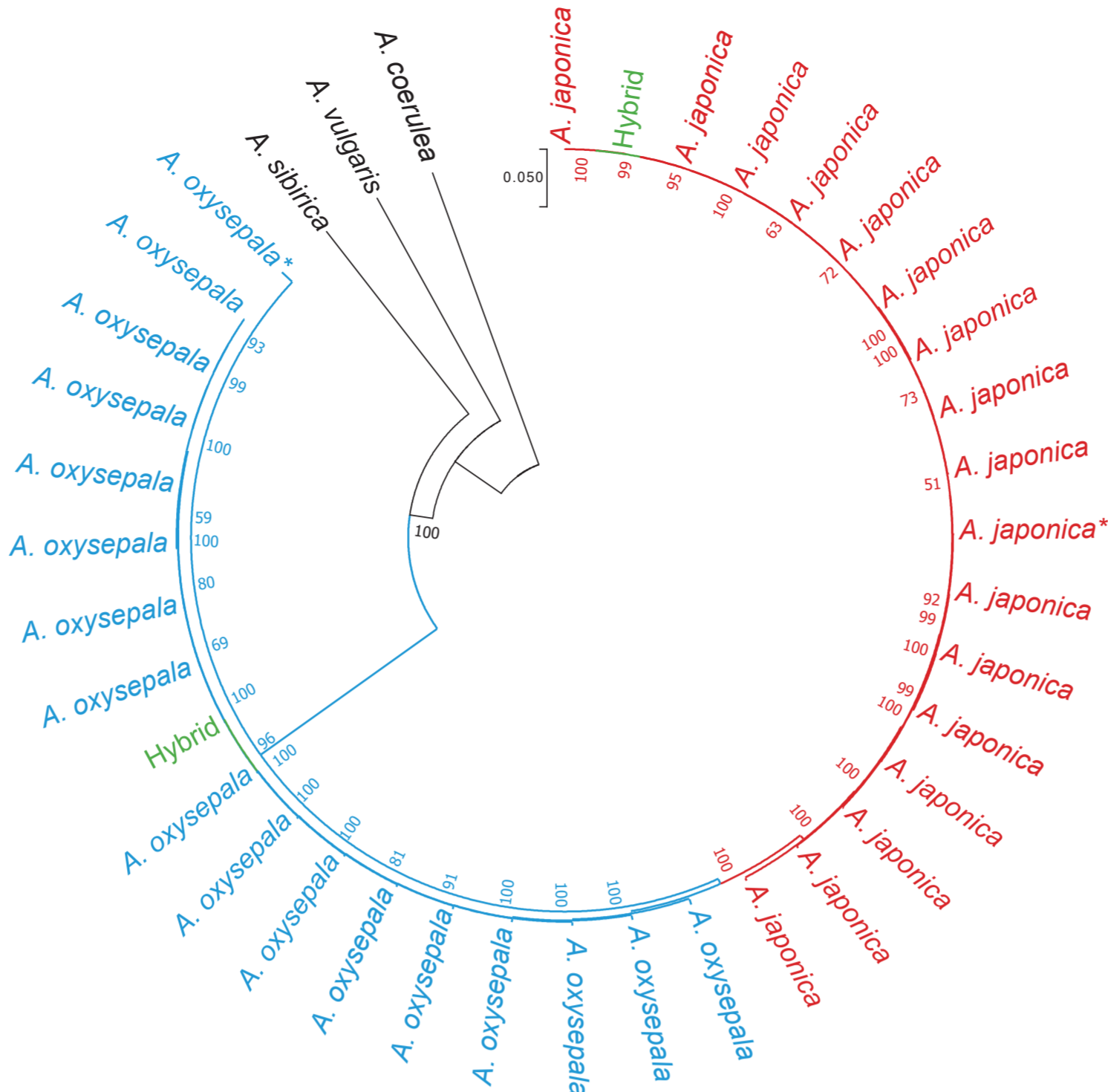

Chr3

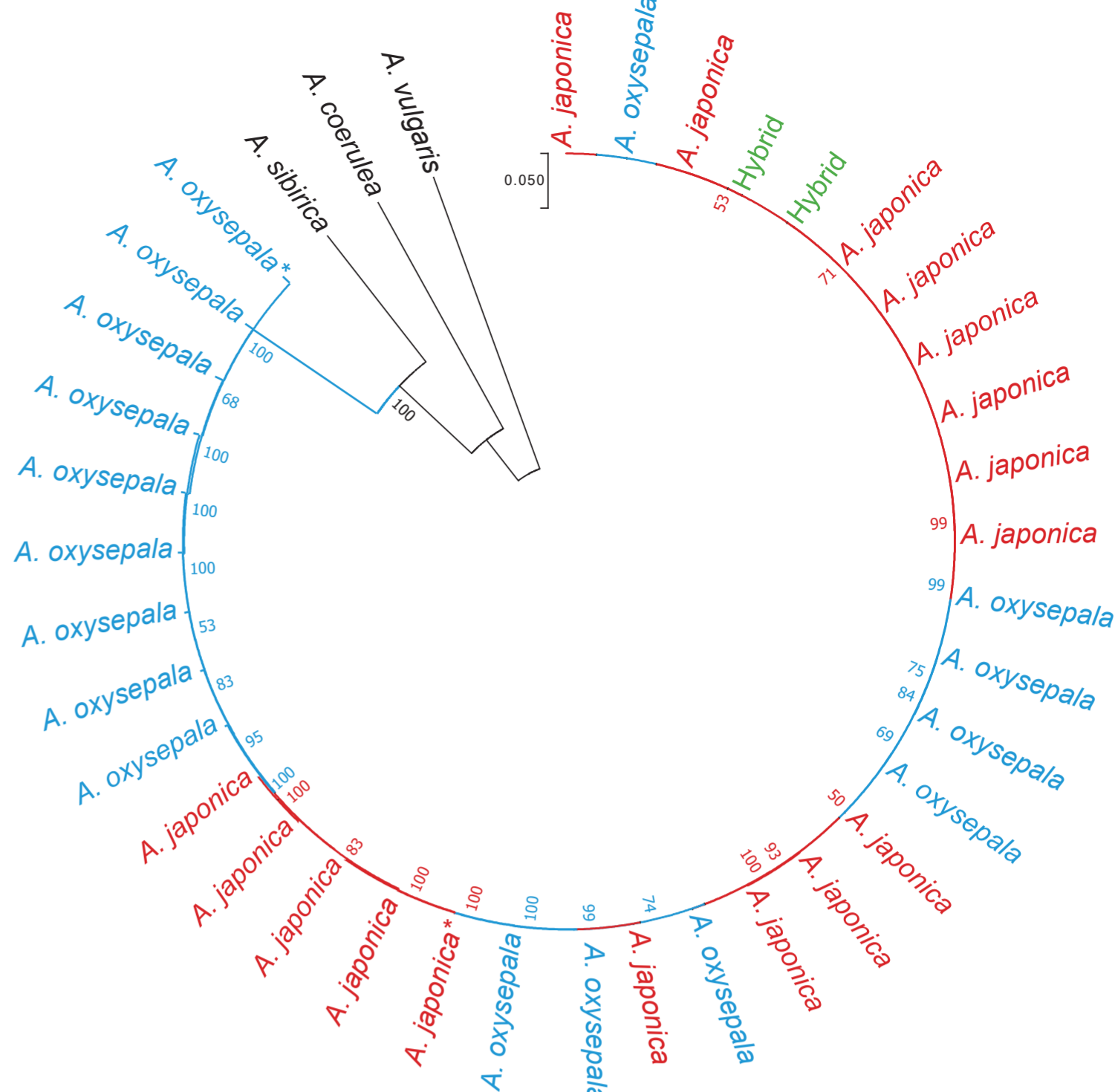

Chr4

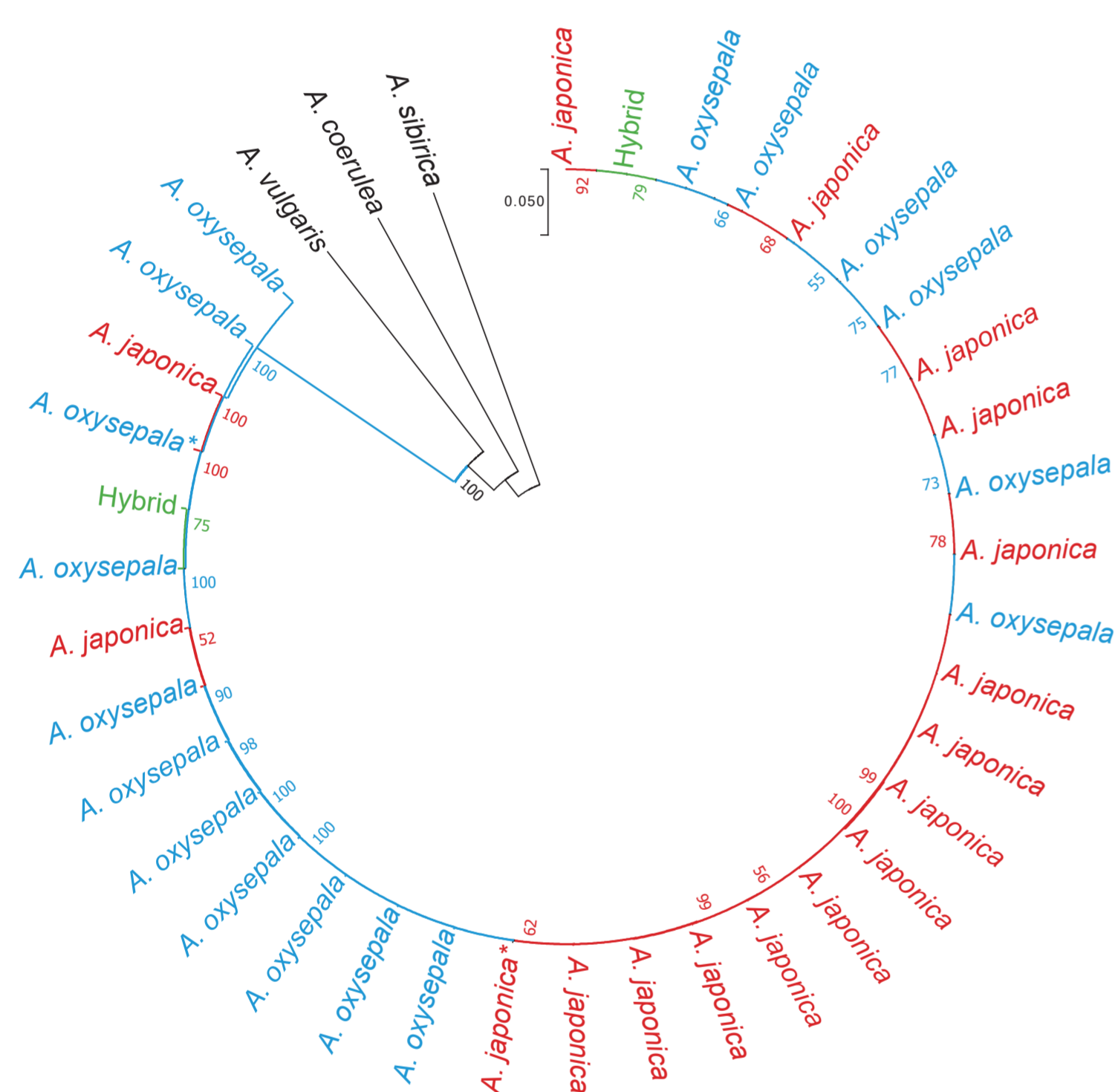

Chr5

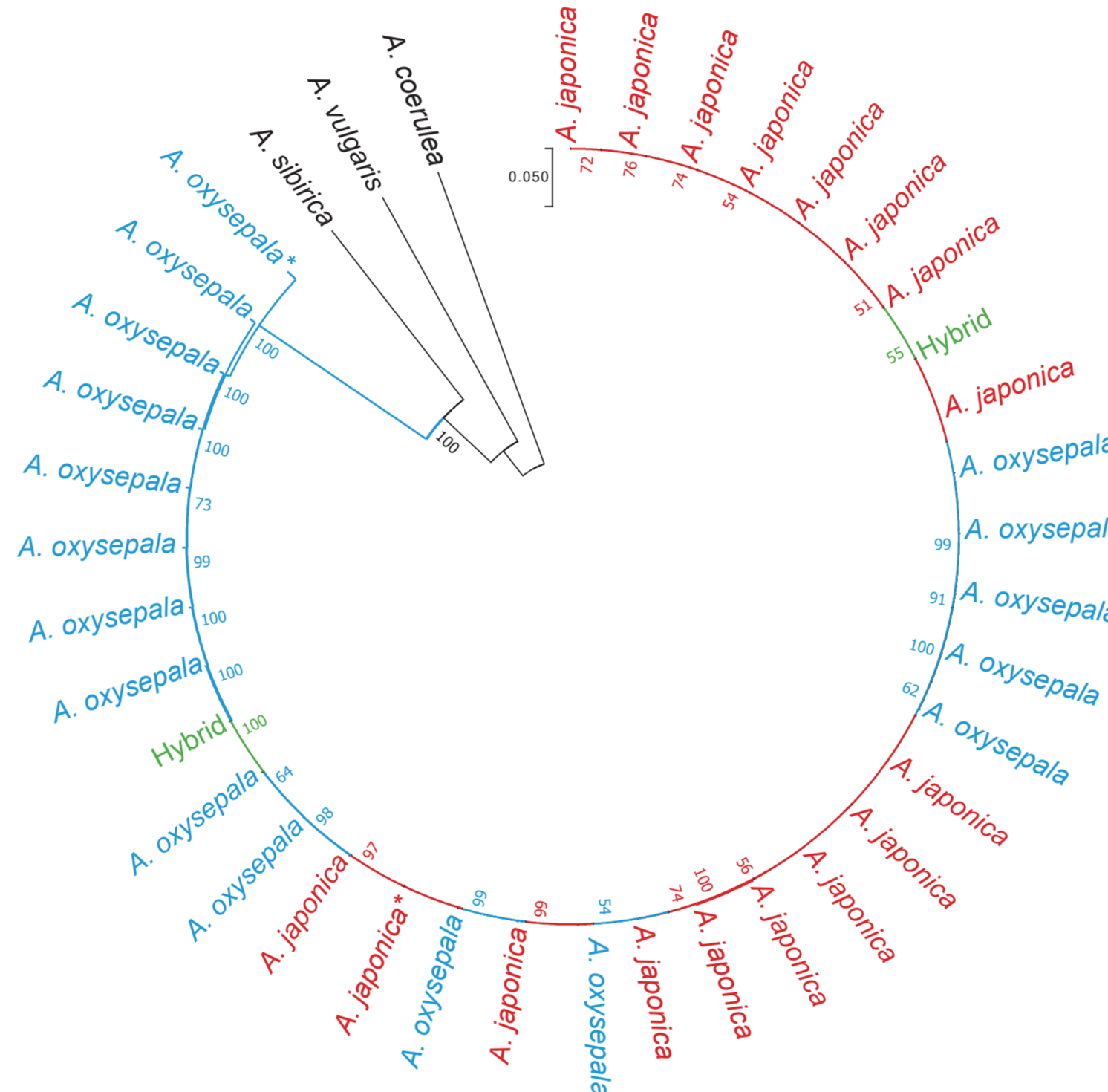

Chr6

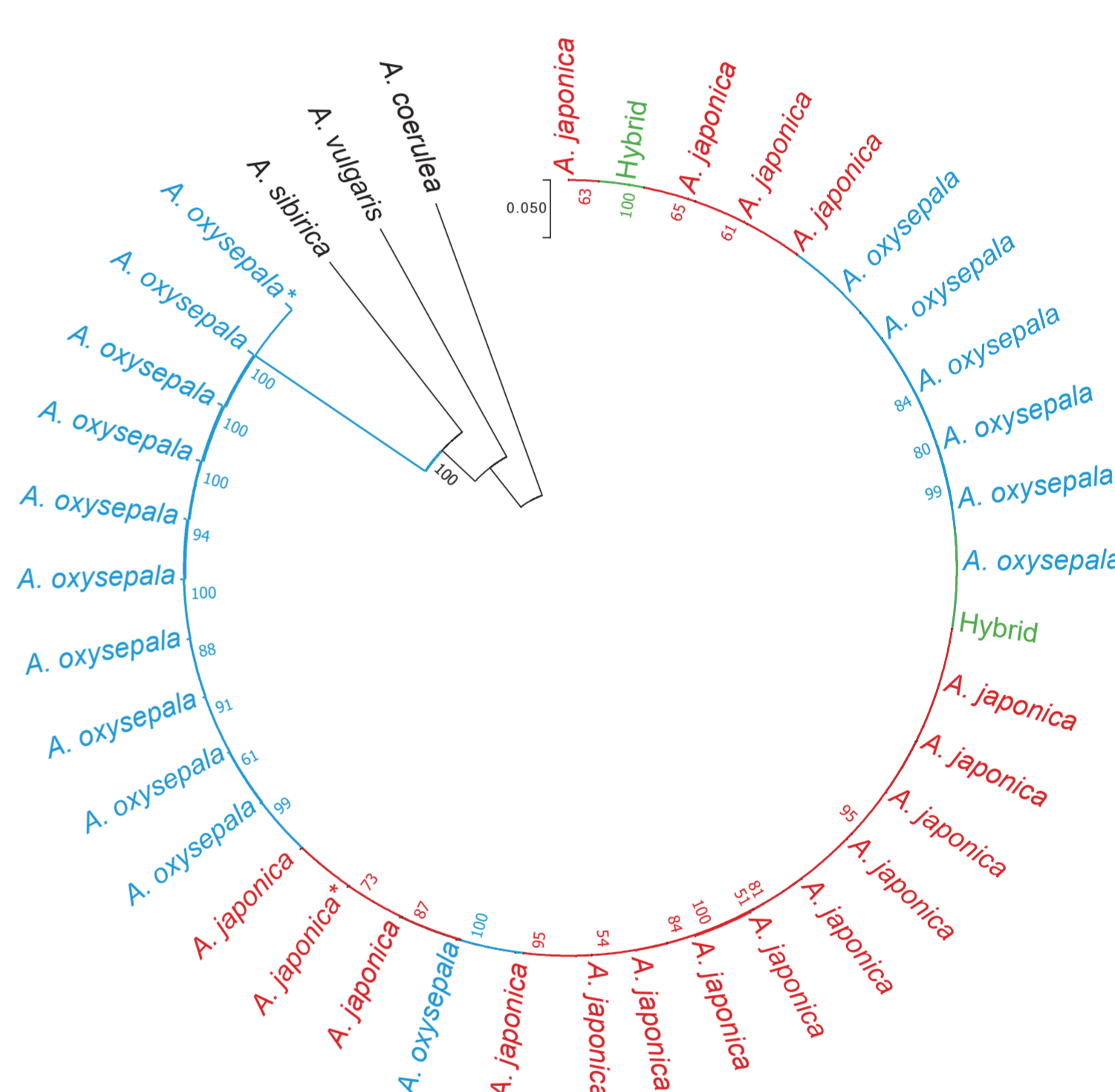

Chr7

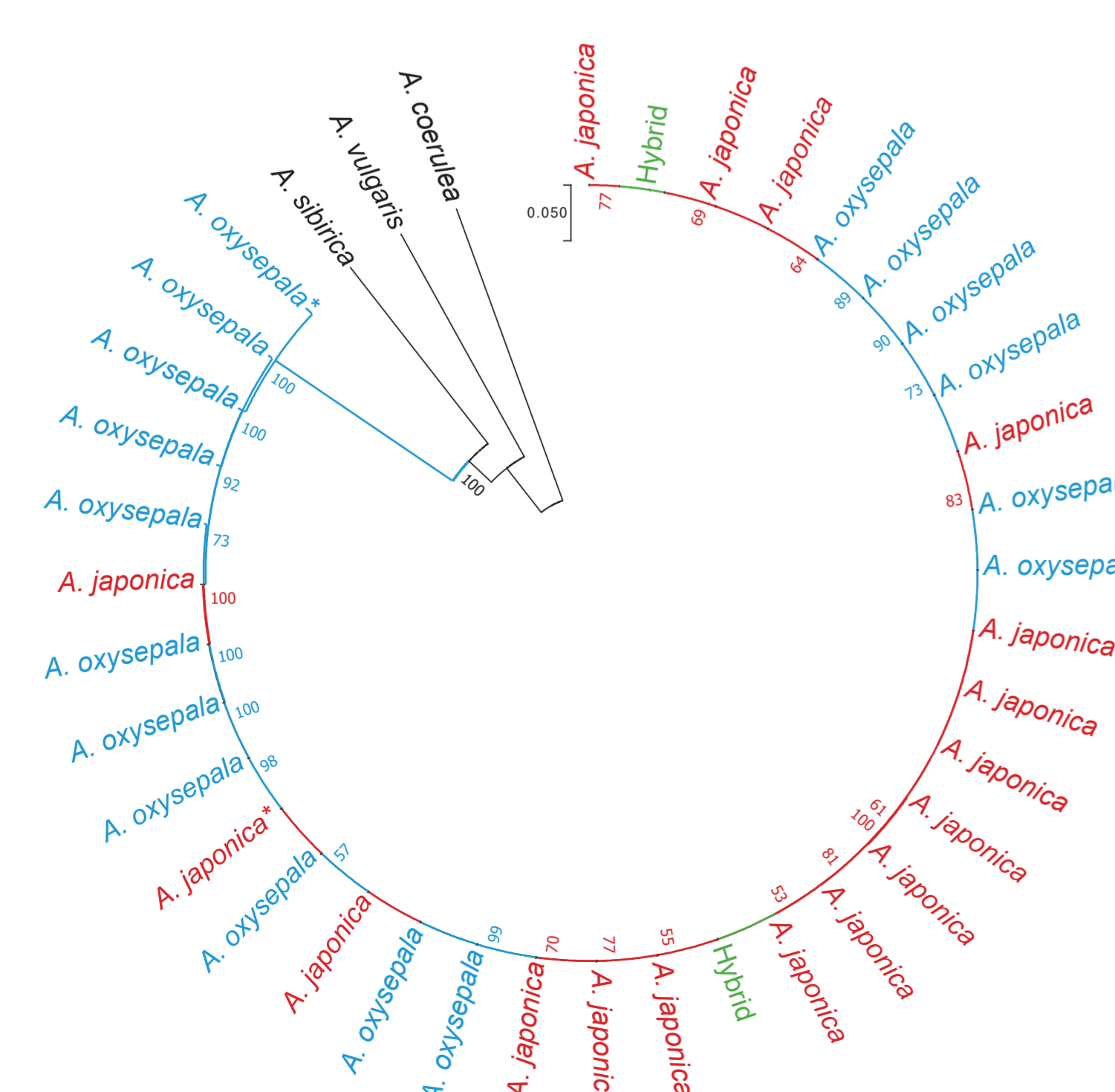

All

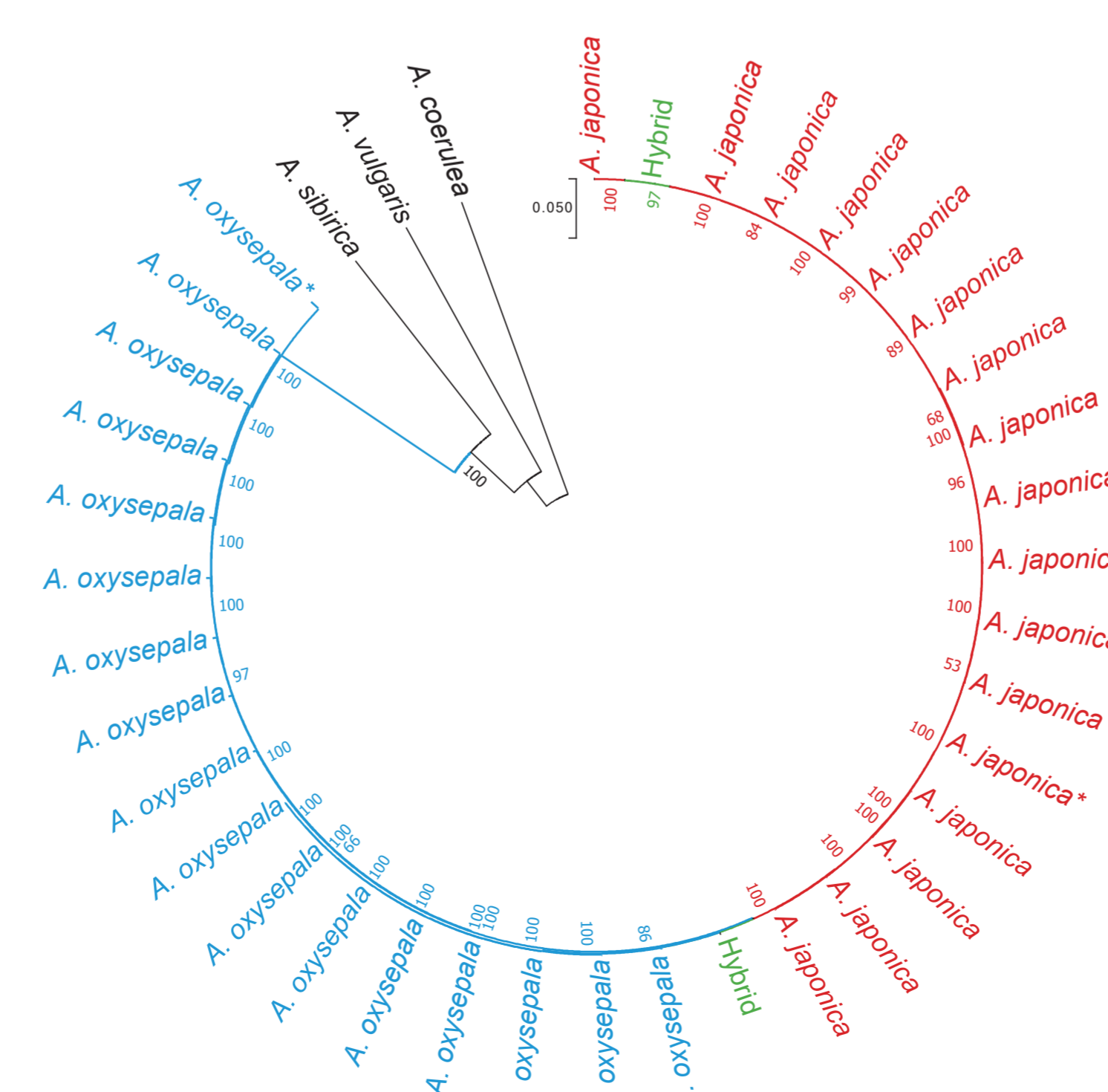

Chloroplast

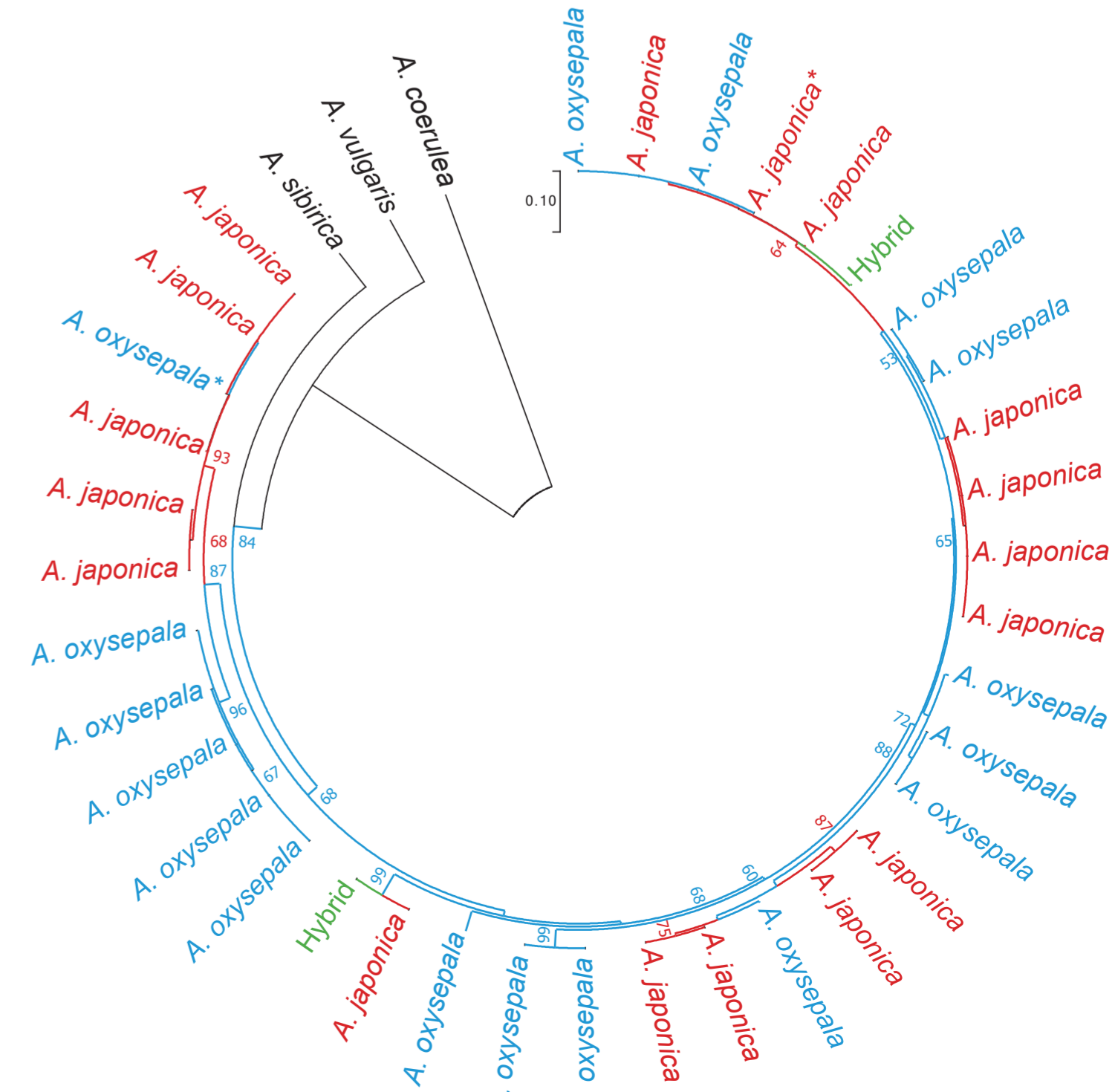

Supplement: Supplementary Data [file evz038_supp.zip › Fig. S4.pdf]

A

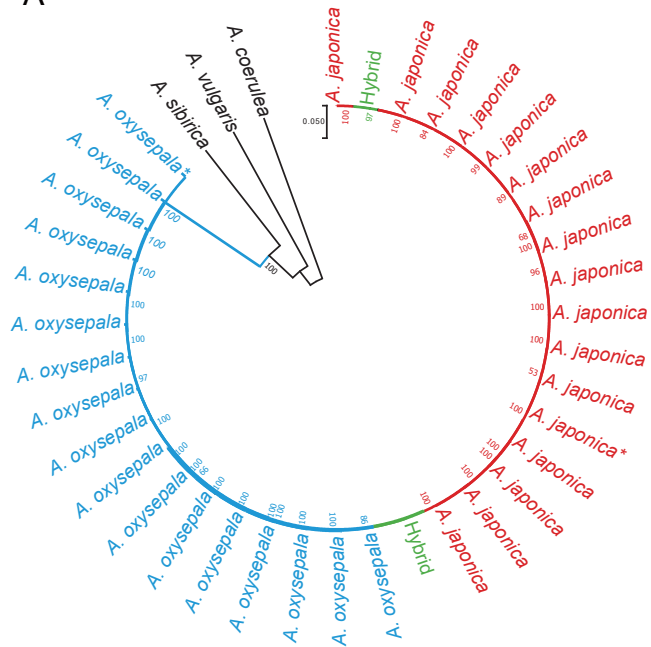

B

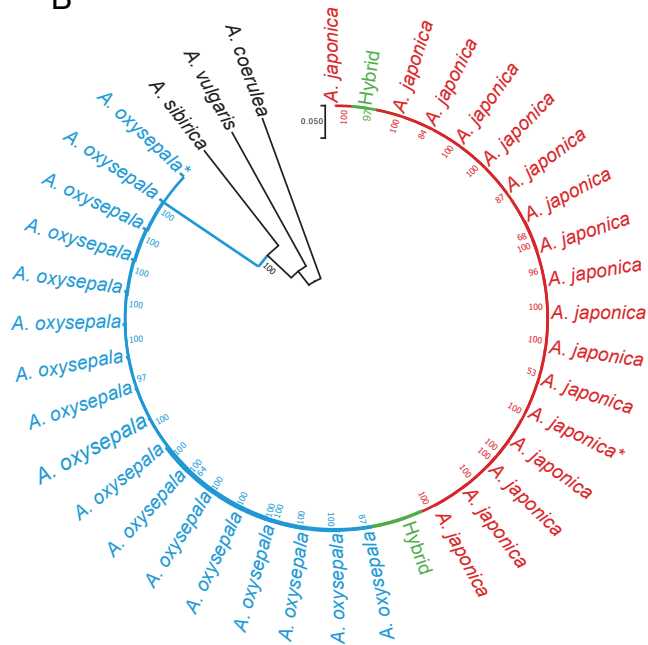

Supplement: Supplementary Data [file evz038_supp.zip › Fig. S5.pdf]

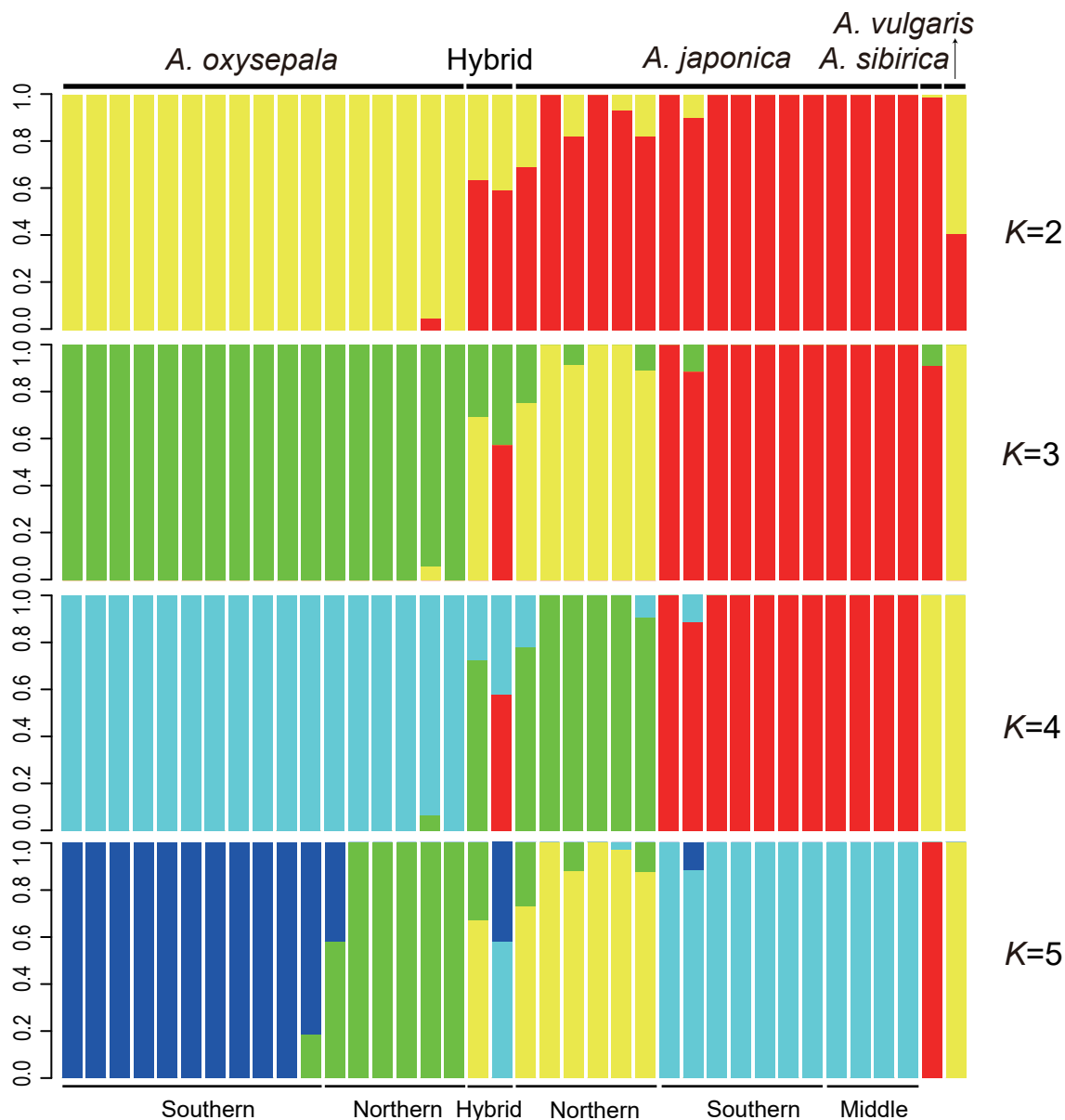

Supplement: Supplementary Data [file evz038_supp.zip › Fig. S6.pdf]

$F_{ST}$  value

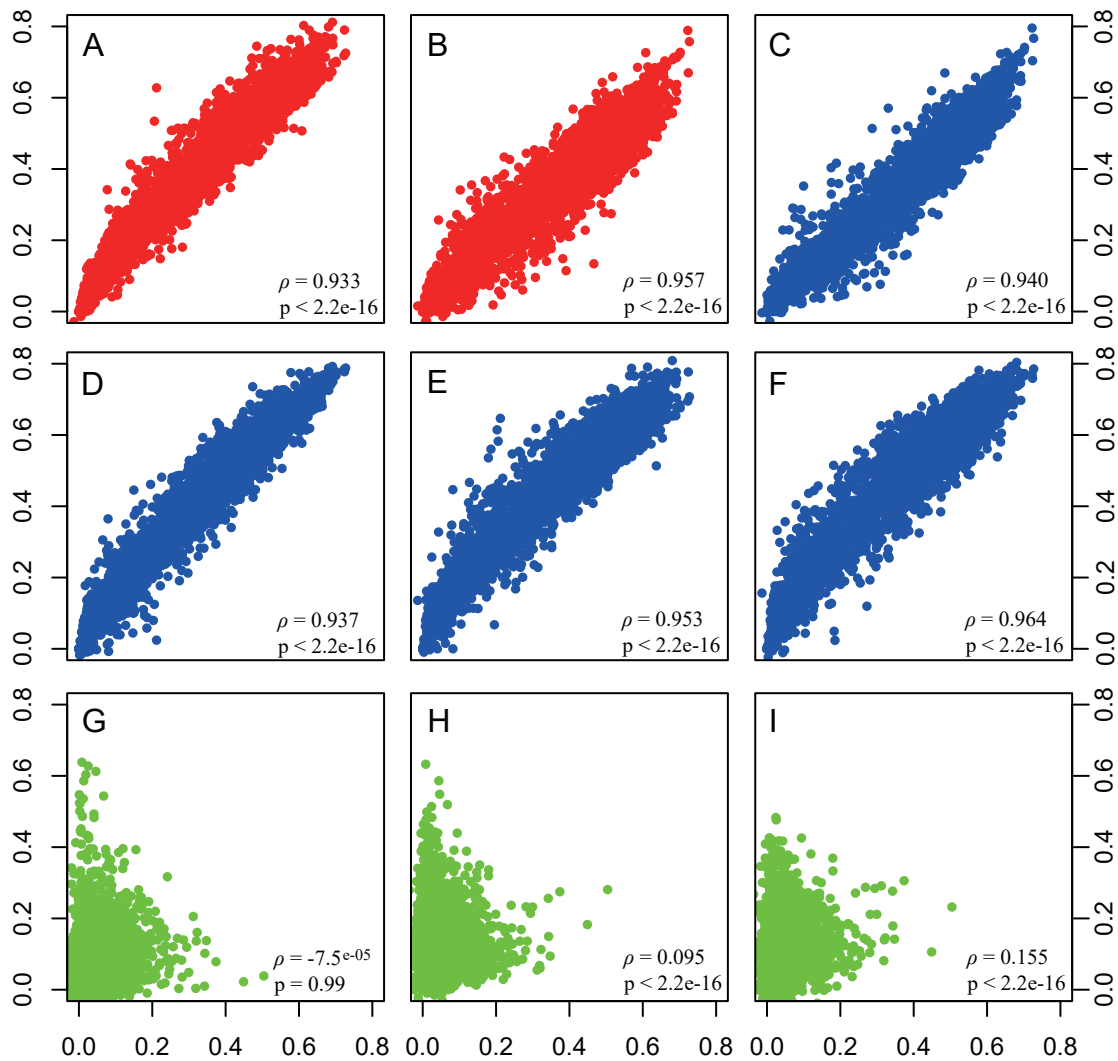

$F_{ST}$  value

Supplement: Supplementary Data [file evz038_supp.zip › Fig. S7.pdf]

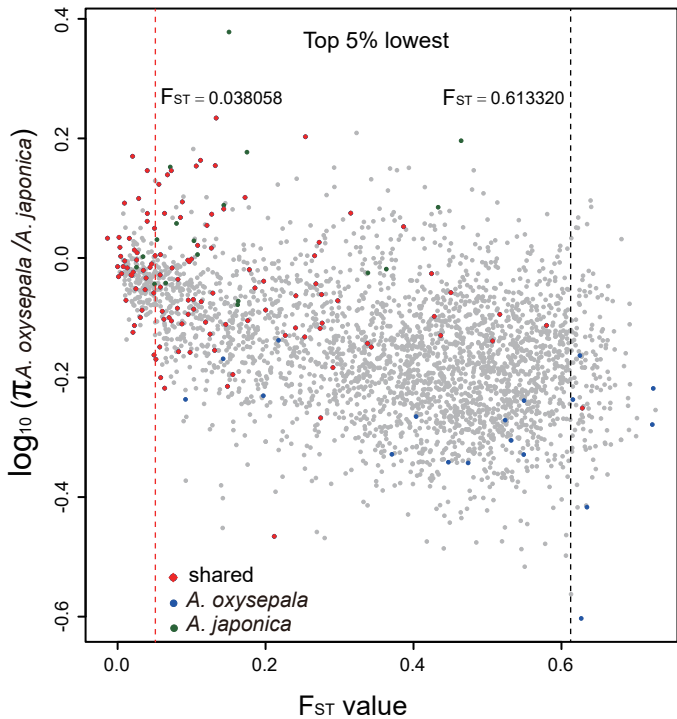

Supplement: Supplementary Data [file evz038_supp.zip › Fig. S8.pdf]

A

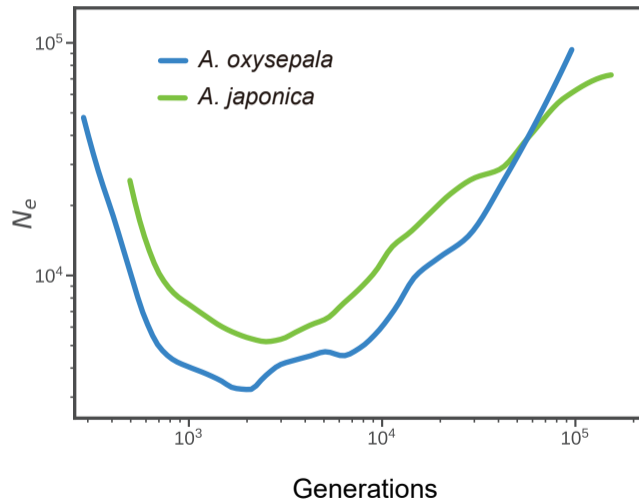

B

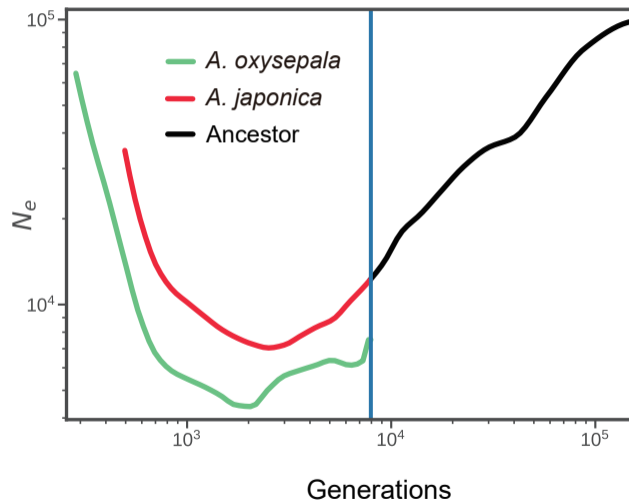

Supplement: Supplementary Data [file evz038_supp.zip › Fig. S9.pdf]
